# Defect Electrochemistry in Stabilizing Corrugated Layered NaMnO_2_

**DOI:** 10.1021/jacs.5c19128

**Published:** 2026-01-29

**Authors:** Shinichi Kumakura, Yusuke Miura, Kei Kubota, Ryoichi Tatara, Eun Jeong Kim, Huu Duc Luong, Yoshitaka Tateyama, Yoshinobu Miyazaki, Tomohiro Saito, Shinichi Komaba

**Affiliations:** † Department of Applied Chemistry, 26413 Tokyo University of Science, Shinjuku, Tokyo 162-8601, Japan; ‡ Research Center for Energy and Environmental Materials (GREEN), 52747 National Institute for Materials Science, 1-1 Namiki, Tsukuba, Ibaraki 305-0044, Japan; § Institute of Integrated Research (IIR), 13290 Institute of Science Tokyo, Midori, Yokohama 226-8501, Japan; ∥ Tsukuba Satellite Laboratory, 201136 Sumika Chemical Analysis Service (SCAS), Ltd., Tsukuba, Ibaraki 305-8565, Japan

## Abstract

Lattice defects in layered metal oxides critically influence the structural stability and electrode reversibility in rechargeable batteries. However, the role of these defects remains poorly understood. Corrugated-layered β-NaMnO_2_ provides an ideal model system because stacking fault (SF) formation plays a key role in its thermodynamic stability. Controlling the SF distribution thus offers a unique opportunity to elucidate the interplay between defects and electrochemical performance. Herein, we show that the partial substitution of Mn with Cu or Zn effectively modulates SF formation in β-NaMnO_2_. Synchrotron X-ray diffraction, scanning transmission electron microscopy, and Raman spectroscopy revealed distinct defect structures: the pristine material exhibited ordered SF domains, Cu-substitution stabilized defect-free zigzag stacking, and Zn-substitution introduced randomly distributed SFs. Both doped materials exhibited improved capacity retention, reflecting suppressed evolution of the α-phase defects during cycling. These findings establish a direct link between SF distribution and electrochemical reversibility, highlighting defect engineering as a strategy for designing durable battery materials.

## Graphical abstract

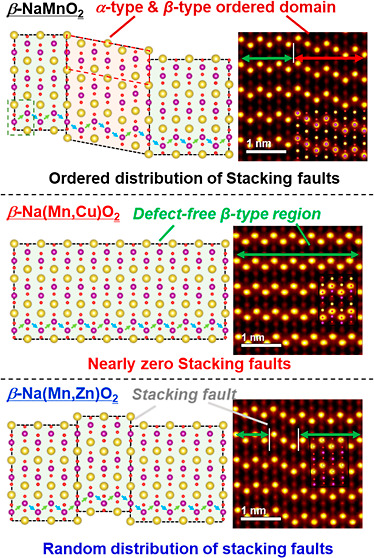

## Introduction

Layered sodium manganese oxides (Na_*x*_MnO_2_, where typically *x* = 0.6–1.0) have emerged as promising cathode materials for sodium-ion batteries (SIBs) owing to their high energy storage potential and absence of rare or critical elements.^1,2^ The growing interest in these materials for energy applications has underscored the need for a deeper understanding of their structural complexity, particularly the lattice distortions and defect chemistry induced by Jahn–Teller active Mn^3+^ ions.^3^ These structural features influence the formation and relative stability of Na_*x*_MnO_2_ phases^4^ including the Na-deficient metastable states,^5,6^ and play a critical role in determining the intrinsic physical properties of Na_*x*_MnO_2_ materials, such as their magnetic and dielectric properties.^7^

Among various layered phases, particular attention has been paid to NaMnO_2_ polymorphs, commonly referred to as α- and β-phases.^8^ The α-NaMnO_2_ phase adopts a monoclinic structure (*C2*/*m* space group) characterized by planar MnO_2_ layers (Figure 1a), whereas the β-NaMnO_2_ phase crystallizes in an orthorhombic structure (*Pmmn* space group) featuring zigzag MnO_2_ slabs (Figure 1b).^9^ Because of their closely overlapping thermodynamic stability, these two structures tend to coexist, with their boundaries serving as lattice defects. The phase competition between α- and β-phases was first experimentally reported in the 1970s^8^ and then was confirmed in 2018 using density functional theory (DFT) calculations.^10^ Owing to this phase competition, the obtained β-NaMnO_2_ often contains a large number of stacking faults (SFs). Most of these faults form along the *bc*-slip plane, forming planar α-type domains (Figure 1c). These structural features have garnered considerable attention in solid-state chemistry research and have been extensively investigated using advanced characterization techniques such as NMR spectroscopy^11^ and scanning transmission electron microscopy (STEM).^12^

**Figure 1:**
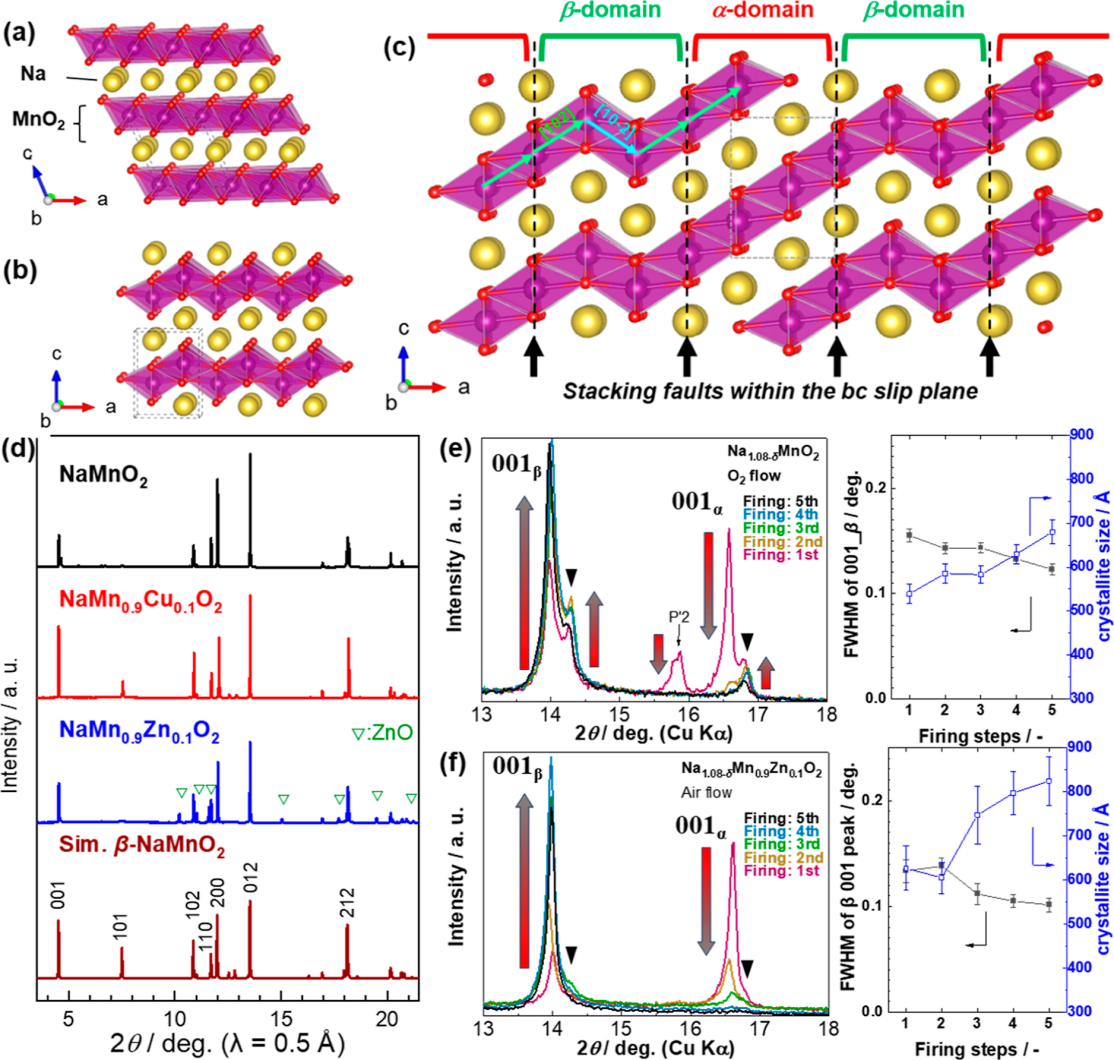
Schematic crystal models of (a) monoclinic α-NaMnO_2_ and (b) orthorhombic β-NaMnO_2_ along the plane perpendicular to the *b*-axis. Na, Mn, and O atoms are represented by yellow, violet, and red spheres, respectively. MnO_6_ octahedra are shown in light purple and extend as a MnO_2_ sheet. (c) Schematic of the intergrowth phase created by the stacking faults observed in NaMnO_2_. (d) Synchrotron XRD patterns of NMO, NMCO, and NMZO. XRD patterns and changes of FWHM_001_ and crystallinity calcined from 1 to 5 times for (e) NMO under O_2_ flow and (f) NMZO in air. Black triangles represent the ordered phase which will be discussed in Figure 3.

Since Billaud et al.’s pioneering studies on high-quality β-NaMnO_2_ with large reversible capacities in SIBs,^13^ extensive efforts have been devoted to optimizing its electrochemical performance.^14–19^ However, phase competition between the α- and β-polymorphs often leads to the formation of SFs or even mixed-phase domains, complicating the evaluation of the intrinsic electrochemical behavior of the pure β-phase. In 2018, our group reported both experimental and theoretical evidence that substitution of Mn with other metals can strongly modulate the phase competition between the α- and β-phases.^10^ DFT calculations of the formation energies of NaMn_0.9_Me_0.1_O_2_ compounds (Me = Mg, Al, Sc, Ti, V, Cr, Mn, Fe, Co, Ni, Cu, and Zn), combined with X-ray diffraction (XRD) analyses under optimized synthesis conditions, revealed the unique stabilization of the β-phase induced by Cu-substitution. Furthermore, our group recently revealed a structural transition mechanism enabled by the deliberate removal of stacking faults, which otherwise hinder the unique slab gliding behavior of the corrugated MnO_2_ layers.^20^ This unique effect of Cu-substitution is attributed to the Jahn–Teller activity of Cu^2+^ (3d^9^), which promotes cooperative lattice distortions. Notably, such distortions were more pronounced in the β-phase than in the α-phase, suggesting that Cu incorporation selectively stabilized this polymorph. However, as that study focused exclusively on SF-free structures, the mechanistic understanding of the influence of SFs on the electrochemical reactions in β-NaMnO_2_ remains elusive.

In this study, we used doping with Zn^2+^ (3d^10^), which is a neighbor of Cu^2+^ (3d^9^) in the periodic table and has a similar ionic radius, to perform a systematic investigation of the electronic and structural effects associated with the Jahn–Teller activity. In fact, according to DFT calculations,^10^ Zn^2+^ doping appears to only relatively weakly affect the phase competition, suggesting that it plays a different role in SF formation than Cu^2+^. In addition, we revisited the synthesis conditions for undoped β-NaMnO_2_ because we found that not only metal substitution but also the synthesis conditions play a crucial role in phase stability and SF formation. Abakumov et al. previously proposed the existence of an incommensurate phase attributed to the nonperiodic distribution of SFs,^12^ and its unique magnetic and dielectric properties were discussed by Orlandi et al.^7^ This incommensurate structure is also expected to be highly sensitive to the synthesis conditions, further complicating the structural and functional characterization of β-NaMnO_2_. The use of different synthesis conditions enabled us to characterize the mechanisms underlying the formation and distribution of SFs, and their impact on the structural stability of β-NaMnO_2_ in SIBs.

Understanding the formation and distribution of SFs is essential for the elucidation of the role that they play in the structural stability and electrochemical performance of SIBs. Therefore, in this study, we systematically investigated how the initial SF-containing crystal structure governs electrochemical activity and degradation pathways during repeated sodium extraction and insertion. By correlating the structural evolution with the electrochemical behavior, we sought to reveal the fundamental mechanisms by which SFs influence the capacity retention and long-term cycling stability in SIBs.

## Results and Discussion

### Synthesis of β-Phase NMO, NMCO, and NMZO Materials

The nondoped, Cu-doped, and Zn-doped samples, NaMnO_2_, NaMn_0.9_Cu_0.1_O_2_ and NaMn_0.9_Zn_0.1_O_2_, were synthesized via solid-state reactions (Hereafter, NMO, NMCO, and NMZO, respectively. See Supporting Information for the synthesis details.). Multiple firings followed by intermediate grinding were performed to obtain pure single-phase materials. The Synchrotron XRD patterns of the final products are shown in Figure 1d. Almost all diffraction peaks of the three samples can be indexed to the β-phase with the *Pmmn* space group, indicating successful formation of the zigzag-layered structure as the main phase^20^ (Figure 1b). All samples consist of highly crystalline rod-shaped particles, with length and width of approximately 10 and 2 μm, respectively (Figure S1a–c). In contrast to NMCO, which exhibited no CuO diffraction peaks, NMZO exhibited distinct ZnO diffraction peaks (marked by green triangles in Figure 1d). STEM-EDS analysis further verified that excess Zn beyond its solubility limit formed large Zn-containing agglomerates, whereas the entire 10% Cu was successfully doped (Figure S1d,e). Importantly, the incorporation of a small amount of Zn^2+^ into the NMZO particles is confirmed by STEM analysis in the next section. Notably, P′2-Na_2/3_MnO_2_ has a higher solubility limit for Zn^2+^ ions up to 10%.^21^

The lattice constants of the samples derived from XRD measurements are listed in Table S1. The values for NMO and NMCO are in agreement with the previous reports by Billaud et al.^13^ and our group,^20^ respectively. The increase in the *b* lattice parameter for doped samples confirms that the effective ionic radii of Cu^2+^ and Zn^2+^ are approximately 13% and 14.7% larger, respectively, than those of high-spin Mn^3+^ (*r*(Mn^3+^, high-spin) = 0.645 Å, *r*(Cu^2+^) = 0.73 Å, and *r*(Zn^2+^) = 0.74 Å in the 6-fold coordination),^22^ indicating that Zn^2+^ ions are partially incorporated into the β-phase. These results demonstrate that we have successfully synthesized the undoped, Cu-substituted, and Zn-substituted samples as single-phase β-type structures.

In contrast to NMCO which contains minimal stacking faults owing to the unique selective β-phase stabilization effect of the doped Cu ions,^10^ NMO and NMZO are prone to forming coexisting α and β phases under the synthesis conditions used in this study. The phase evolution of NMO and NMZO during repeated calcinations provided important insights into the characteristics of the phase mixture. First, the influence of the calcination atmosphere was investigated. The crystal structures of the NMO powders varied significantly depending on the firing atmosphere (air, oxygen, or argon) (Figure S2). The air-calcined NMO exhibited a mixed phase of α-NaMnO_2_ and β-NaMnO_2_ where the intensity of the 001 diffraction from the β-phase at 13.9° (*I*_001β_) was lower than that of the α-phase at 16.5° (*I*_001α_ > *I*_001β_). The O_2_-calcined NMO showed a three-phase mixture of α-phase, β-phase, and P2/P′2-type phases because additional reflections at 15.7° suggests a formation of P2- or P′2-Na_2/3_MnO_2_.^6^ The formation of Na-deficient P2-type phases is attributed to higher Na volatility in higher oxygen partial pressure and simultaneous oxidation of Mn­(III) to Mn­(IV) under O_2_.^23^ Nevertheless, the β-phase is favored compared to the α-phase (*I*_001α_ < *I*_001β_) in oxygen. By contrast, single-phase α-NaMnO_2_ was obtained under Ar flow with only a trace amount of β-phase (*I*_001α_ ≫ *I*_001β_). Thus, oxygen atmosphere was selected for the subsequent synthesis and structural characterization of NMO.

The impact of the calcination atmosphere on phase formation was significantly altered in the presence of copper dopants. The NMCO pellets predominantly formed the P2-type phase when calcined in O_2_ atmosphere (Figure S3). This observation further supports the hypothesis that sodium volatility and the oxidation of manganese are markedly enhanced in O_2_ compared with air. In Ar, CuO remained unreacted, inducing a significant number of SFs. Calcination in air appears to be the optimal condition for NMCO, as the high oxygen partial pressure stabilizes phase formation while simultaneously accelerating Na loss, Mn-oxidation, and CuO formation.

As a single calcination did not yield single β-type phase for NMO and NMZO compositions, this study proposes that a multistep calcination process is essential for obtaining a well-defined β-phase. Figure 1e,f illustrate the phase evolution through five calcinations (first to fifth in the figure) by repeating the same procedure including heating to 1050°C and quenching to RT for NMO and NMZO in O_2_ and in air, respectively. (See the XRD patterns in the wider 2θ range in Figure S4.). The O_2_-calcined NMO exhibits a noticeable enhancement in β-phase purity immediately after the second calcination as evidenced by the distinct decrease in the α- and P2-related signals (highlighted by red arrows). After five calcinations, the phase purity and crystallinity of the β-phase are continuously enhanced, yielding crystallite sizes of as high as 680 Å. Importantly, the diffraction peaks at the 2θ angles of 14.3° and 16.8° (highlighted by black triangles) remained and even intensified with successive calcinations. We discuss the origin of these signals in the next section and propose that these peaks can be attributed to the formation of ordered α- and β-stacking. For NMZO, the 001_α_ reflection disappeared after the third calcination steps. The 001-crystallite size increased from 620 Å to 825 Å after five air calcinations, achieving highly crystalline β-phase while ZnO reflections remained. Altering the Zn concentration was ineffective in suppressing the formation of the α-phase (Figure S4c), which further motivated the adoption of the multicalcination approach to control SF distribution.

Synthesis conditions were optimized for each dopant, and the difference is attributed to their electronic structures rather than ionic size. Zn doping does not significantly alter the intrinsic α-β phase competition, similar to the undoped sample; therefore, multiple calcination steps are required to stabilize the β-phase, which exists as a high-temperature phase.^24^ In contrast, Cu^2+^ ions exhibit Jahn–Teller distortion, strongly stabilizing the β-phase^10^ and allowing single-step calcination. These results experimentally demonstrate that this competition can be modulated by altering the calcination atmosphere and the nature of the substituent species. Our previous work^10^ examined various 3d metal dopants and their impact on phase stability; however, the synthesis was limited to a single calcination step. When multiple calcination steps and atmospheric control are optimized, the underlying mechanism of SF formation, such as local distortion pinning and charge randomness, is expected to apply to other dopants. Future work will explore this universality and elucidate its conncection to crystallographic and magnetic properties.^12,25^

### Defect Distribution in β-NMO via Cu and Zn Doping

Considering that SF formation is regarded as the intergrowth of the α-phase, the amount of SFs and their distribution are strongly affected by the choice of dopants and synthesis conditions. To elucidate the effects of dopants and synthesis conditions on the formation and distribution of SFs, we carefully investigated the local structure, such as the atomic arrangement, by scanning transmission electron microscopy (STEM). Instead of the NaMn_0.9_Cu_0.1_O_2_ sample, NaMn_0.85_Cu_0.15_O_2_ (hereafter, denoted as NMCO-2) was chosen as the specimen for examination by STEM because it has minimal SFs.^20^ Figure 2 shows the selected area electron diffraction (SAED) patterns, high-angle annular dark-field (HAADF) STEM images along [010] and the magnified STEM images for NMO (Figure 2a–c), NMCO-2 (Figure 2d–f), and NMZO (Figure 2g–i), respectively. For all three materials, the main reflections match well the reflections of the β-type orthorhombic lattice. A comparison and indexing of the simulated SAED patterns are shown in Figure S5. NMCO-2 exhibited distinct SAED reflections and no SFs in the STEM images within the in-plane area of 10 × 10 nm^2^ and along the depth direction exceeding 100 nm. The SF-free β-type domain is highlighted with green arrows in Figure 2e,f. The homogeneous Cu distribution in the bulk was also confirmed by STEM-EDS (Figures 2f and S6a). DFT calculations indicate that Jahn–Teller active Cu^2+^ ions preferentially stabilize the β-phase while destabilizing the α-phase, thereby suppressing SF-formation,^10^ which is consistent with SXRD and STEM observations.

**Figure 2:**
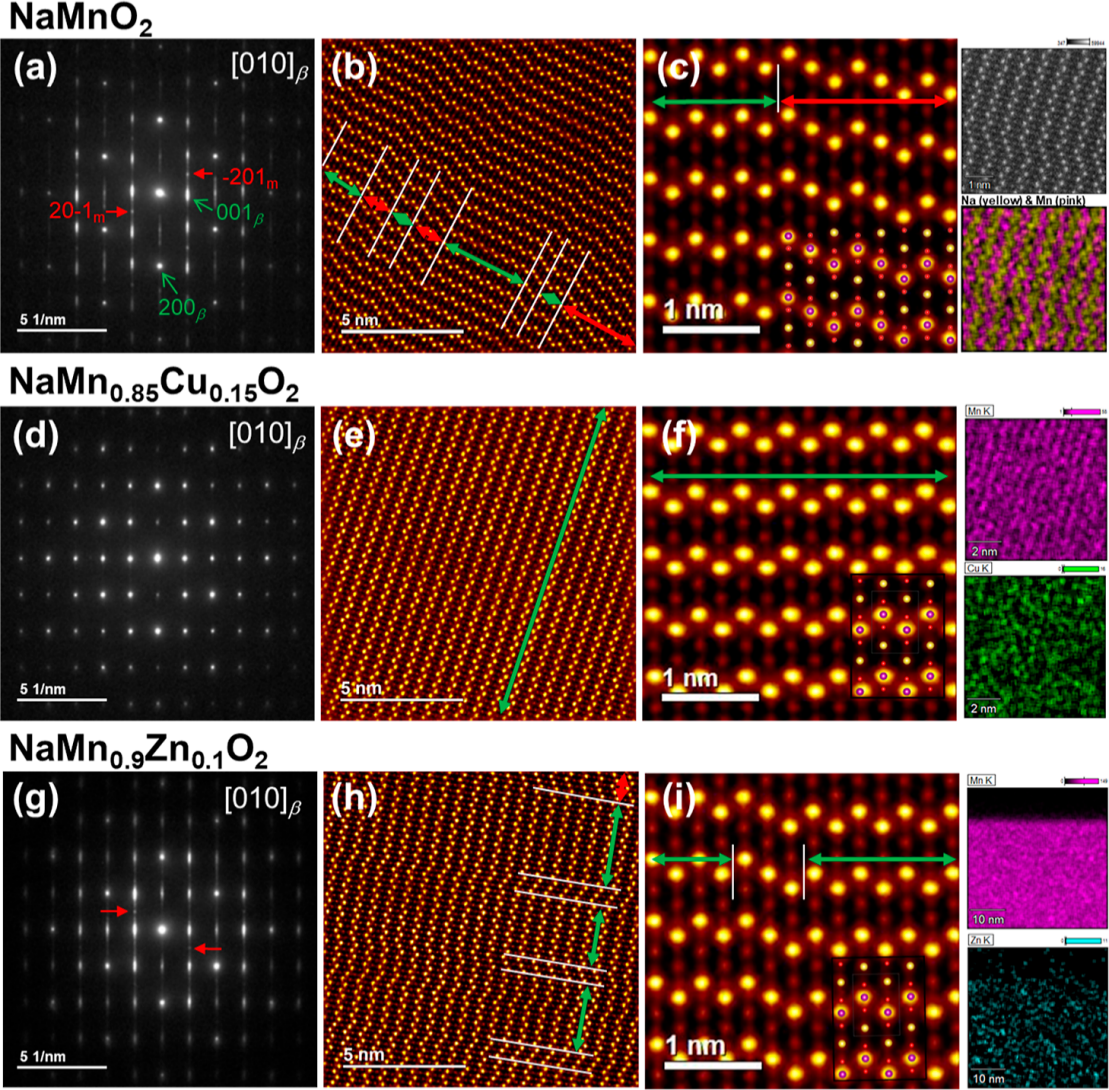
Local structures modified by Cu or Zn-substitution. Selected area electron diffraction (SAED) patterns of (a) NaMnO_2_, (d) NaMn_0.85_Cu_0.15_O_2_, and (g) NaMn_0.9_Zn_0.1_O_2_ along the [010]_β_ axis. Here, “m” represents a monoclinic lattice. A red arrow indicates the diffraction from the modulated monoclinic lattice. High-angle annular dark-field (HAADF) scanning transmission electron microscopy (STEM) images of (b) NaMnO_2_, (e) NaMn_0.85_Cu_0.15_O_2_, and (h) NaMn_0.9_Zn_0.1_O_2_ along the [010]_β_ axis. Green and red arrows indicate the ranges of the orthorhombic and modulated monoclinic domains, respectively. Enlarged HAADF-STEM images with EDS mapping of (c) NaMnO_2_, (f) NaMn_0.85_Cu_0.15_O_2_, and (i) NaMn_0.9_Zn_0.1_O_2_ along the [010]_*β*_ axis. The insets illustrate the atomic arrangement.

By contrast, both NMO and NMZO show diffuse reflections with streaks along the [100] axis in the SAED patterns, which is consistent with the presence of multiple slip planes observed in the STEM images (as marked by the white lines in Figure 2b and h). Based on the high-magnification STEM images, the observed defects appear not only as randomly distributed SFs but also as partially ordered arrangements, that is, the additional SAED spots indicated by red arrows. Here, we propose a superstructure model with a monoclinic unit cell consisting of a β–α–β (*BAB*) ordered domain configuration (Figure 3a). This superstructure features a single α-type intergrown domain sandwiched by two β-type domains. The simulated SAED patterns (Figure S5d) based on the structural model of the proposed *BAB* superstructure (Table S2) show good agreement with the additional spots. In addition to the distinct spots, elliptically distorted diffraction spots, such as the *k*01 spots, indicate the existence of the *BAB* superstructure. The 001_β_ and 001_*BAB*_ diffraction spots were close to each other but were located at different positions; thus, the spots were elongated along *c*-axis (Figure S5c). The unknown diffraction peaks at 14.3° and 16.8° (Figure 1e), highlighted with black triangles) were assigned using the superstructure model. Most of the unassigned peaks in the range 4–9° in the SXRD patterns can be explained and indexed using this model, as shown in Figure 3b. Repeated *BAB* domains (i.e., ...BABABABA···domain) were found in NMO and are indicated by red arrows in Figure 2b,c. Among the previous studies related to the α- and β-NaMnO_2_ polymorph, several reports have considered the distribution of SFs.^12,30^ Abakumov et al. proposed the incommensurate monoclinic phase for β-NaMnO_2_ which was obtained using a synthesis procedure limited to two calcination steps.^12^ Our multistep calcination approach, which extends calcination up to five cycles, enables the formation of highly crystalline commensurate *BAB* domains as verified through STEM observation. Furthermore, by combining STEM with SXRD analysis, we precisely determined the structural parameters of these domains for the first time.

**Figure 3:**
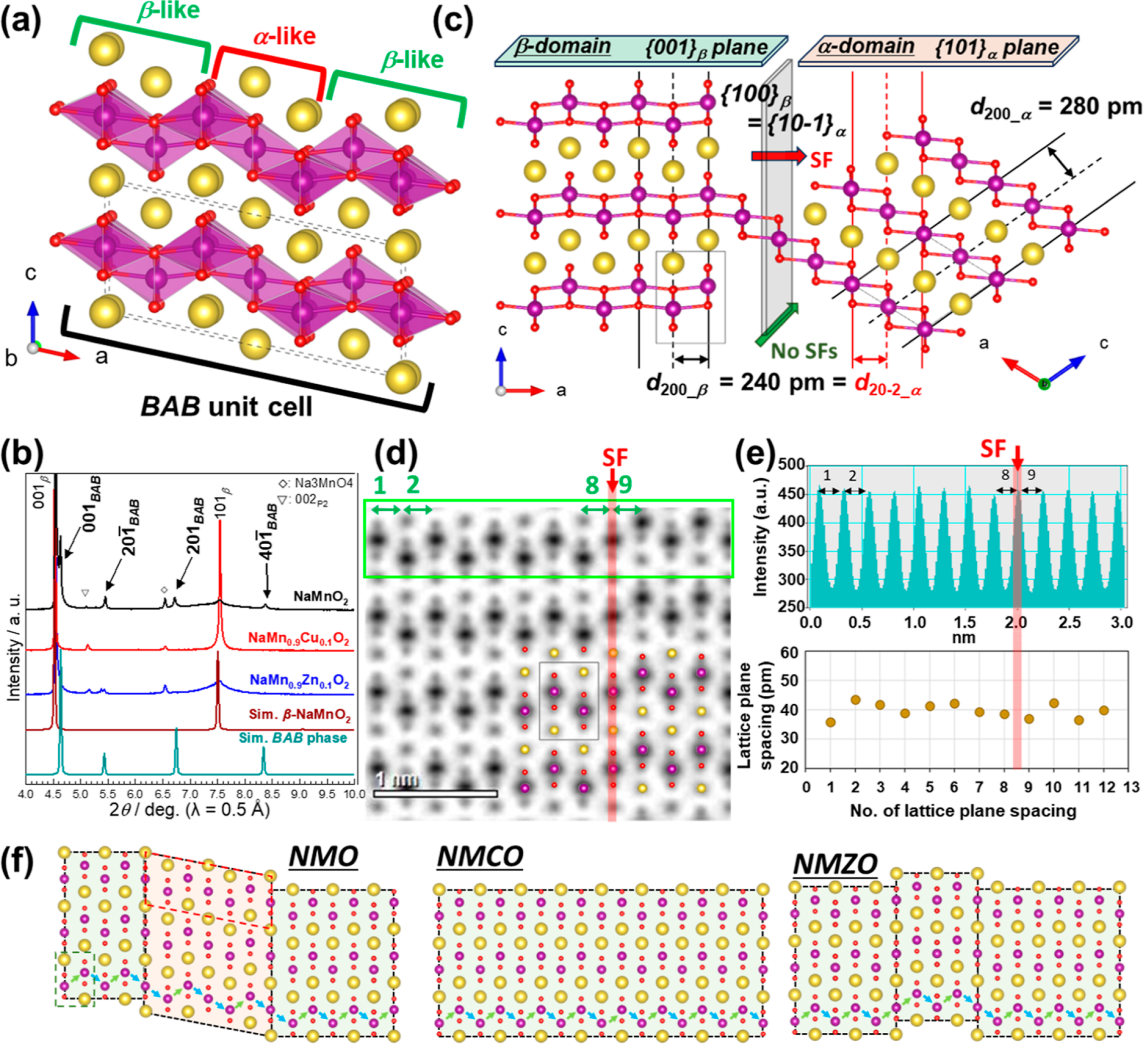
Summary of SF distribution in the β-phase. (a) Schematic crystal model of the monoclinic *BAB* superstructure. (b) Enlarged SXRD patterns of NaMn_0.9_Me_0.1_O_2_ (Me = Mn, Cu, Zn). Simulated XRD patterns of orthorhombic β-NaMnO_2_ and modulated monoclinic NaMnO_2_ are shown as references. ▽ indicates the 002 diffraction from P2-Type and ◊ indicates the diffraction peaks from cubic Na_3_MnO_4_. (c) *d*-spacing for α- and β-polymorphs. ABF-STEM line profiles for NMO: (d) the green box corresponds to the scanned line, and (e) Mn–Mn distance distribution. (f) Schematic of the stacking in the *ac* plane for NMO, NMCO, and NMZO.

Using atomic resolution analysis, we further explored the formation mechanisms of the structural defects from the perspective of local atomic arrangements. The (10–1)_α_ plane is parallel to the (100)_β_ plane with an interplanar spacing of d_200_β_ ≈ d_20–2_α_ ≈ 240 pm (Figure 3c). The spacing was determined by the ABF-STEM line profiles of NMO along the [010]_β_ direction (Figure 3d,e). The peak-to-peak spacing (Figure 3e lower) measured from the horizontal line profile (Figure 3e upper) of the intensity in the green box shown in the ABF-STEM image (Figure 3d), corresponding to the lattice plane spacing, was approximately 240 pm regardless of the presence of SFs, as indicated by the red arrow. This lattice compatibility indicates that the planar slip along the *bc* plane for the β-phase is energetically favorable, facilitating formation of α-type domains. The anisotropic behavior of the SF formation was evident because SFs were minimally present along the *b*- and *c*-axes. Out of several STEM measurements, only a single instance of SF formation along the *b*-axis was observed in NMO, whereas no such faults were detected in NMCO or NMZO. The SF along the *ac*-plane causes local lattice distortions (Figure S7). The lack of observable SFs along the *bc*-plane, even in regions with thickness exceeding 100 nm, which corresponds to more than 300 unit-cell layers, strongly suggests high structural coherence along both *b*- and *c*-axes. STEM analysis along the [100] zone axis for the three samples further verified the highly ordered crystalline phases (Figure S8). The formation energies of the α, β, and *BAB* phases were obtained by DFT calculations and compared (Figure S9, see the calculation detail in SI).^27–34^ It was found that the formation energies of all three phases were highly similar in both undoped and Zn-doped systems, verifying the thermodynamic competition between the phases and supporting the feasibility of *BAB* phase formation.

Unlike NMO, NMZO does not exhibit large *BAB* domains (Figure 2h,i). This is consistent with the subtle additional diffraction peaks in its SXRD pattern and weaker SAED spots related to the superstructure. This indicates that the randomly distributed Zn ions suppress the formation and growth of *BAB* domains. STEM-EDS analysis of NMZO confirmed the successful incorporation of Zn into the host structure (inset of Figure 2i); the Zn content was approximately 1 mol % relative to Mn (Figure S6b). Zn doping exhibits only a marginal effect in mitigating SF formation at the doping level achieved in this study. Although the solubility limit may be extended through optimization of synthesis parameters (e.g., prolonged dwell times or the use of more reactive Zn precursors), DFT calculations at 8.3% Zn indicate persistent α-β phase competition both in this study (Figure S9) and the previous sttudy.^10^ This suggests that further increases in Zn content would exert minimal influence on SF suppression. Nevertheless, variations in SF distribution as a function of Zn concentration remain noteworthy, as they may have consequential implications for electrochemical performance.

Figure 3f displays the schematic illustration of the differences in the SF sequence for the β-type products with different doping. Although both NMO and NMZO exhibit a certain amount of SFs, their distribution patterns differ significantly. In NMO, the SFs tend to form ordered α-domains, giving rise to the *BAB*-type superstructure, whereas in NMZO, the SFs are randomly distributed with no long-range ordering. By contrast, NMCO retains a nearly SF-free structure, rendering it an important counterpart material for comparison. Thus, we found that β-NaMnO_2_ can assume two different stacking-fault structures or a fault-free structure; these structures can be controlled through calcination and doping strategies due to their thermodynamic proximity as supported by DFT calculations.

### Defect Impact on the Local Structure via Raman Analysis

Raman spectroscopy offers a powerful means of probing the local crystal structure. It provides complementary insights into the average structure obtained from the diffraction and microscopic analyses discussed in the previous sections. Thus, the combination of XRD/STEM analyses with Raman spectroscopy measurements enables a comprehensive understanding of lattice distortions and defects in β-phase materials.

The ideal β-NaMnO_2_ crystallizes in the *Pmmn* orthorhombic space group, which belongs to the *D*_2*h*_ point group. In this structure, the unit cell contains one Na atom and one Mn atom at the 2b Wyckoff position and two O atoms at the 2a position. Based on group theory,^35^ β-NaMnO_2_ is expected to show 12 Raman-active modes: 4A_g_ + 4B_2g_ + 4B_3g_.

Figure 4a,b present schematic illustrations of the representative Raman modes and the corresponding Raman spectra of pristine NMO, NMCO, and NMZO..

**Figure 4:**
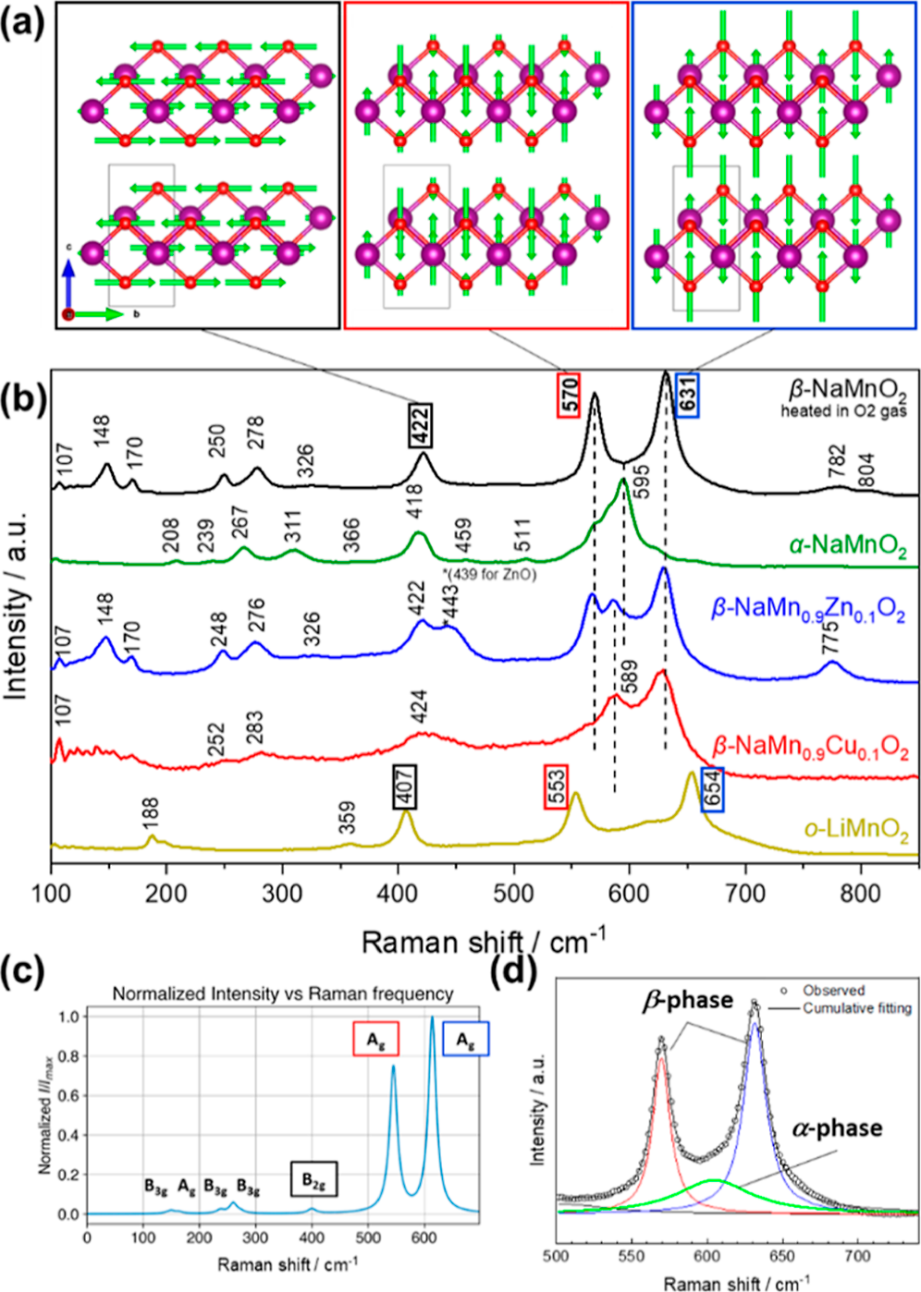
(a) Main Raman modes for the β-phase, corresponding to two Mn–O stretching modes at 631 and 570 cm^–1^, and one bending mode at 422 cm^–1^. (b) Raman spectra of pristine samples compared with the reference materials (α-NaMnO_2_ and *o*-LiMnO_2_). (c) Raman spectrum for the ideal β-NaMnO_2_ simulated using the structure obtained by DFT calculations, and (d) the fitting result of the Raman spectrum for NMO.

For comparison, the obtained spectra are shown together with those of α-NaMnO_2_ ^36^ and orthorhombic LiMnO_2_,^37^ which share the same crystal structure as β-NaMnO_2_. Three representative Raman modes are observed for both β-NaMnO_2_ and LiMnO_2_. For NMO, the two prominent peaks at 570 and 631 cm^–1^ are attributed to the Mn–O stretching modes.^35,38–40^ The peak at 422 cm^–1^ corresponds to the Mn–O bending mode (Figure 4a). The Raman peaks observed below 400 cm^–1^ in the spectra are more complex due to the additional vibrational contributions from the NaO_6_ octahedra.^39^ The Raman peak assignments were verified using simulated Raman spectra calculated based on the structural model obtained by DFT calculations. Figure 4c presents the simulated spectrum for the ideal β-NaMnO_2_ structure. Harmonic phonon calculations were performed using the Phonopy code^41,42^ to identify the vibration frequencies and mode eigenvector at the Γ points. The phonon density of states and the expected Raman modes of this structure are provided in the Supporting Information (Figure S10b and Table S3). The Raman activities were obtained using the Phonopy-Spectroscopy script.^43^ It is observed that the simulated peaks agree with those in the experimental spectrum of NMO. Three distinct peaks were used for the subsequent phase identification. α-NaMnO_2_ shows a prominent peak at 595 cm^–1^, which can be assigned to the A_g_-type Mn–O stretching modes based on the simulation (Figure S10a and Table S4). Although the same simulation methodology was applied to the *BAB* structure, the resulting pattern closely resembled that of the β-phase, with only slight peak shifts that may not be distinguishable in the experimental spectra (Figure S10c and Table S5). Main Raman modes and simulated Raman spectra for α-phase and *BAB*-phase are shown in Figures S11 and S12, respectively. Based on the spectral features and peak assignments discussed above, peak fitting was performed for the Raman spectrum of NMO as shown in Figure 4d. The experimental spectrum between 500 and 700 cm^–1^ was successfully reproduced by incorporating the stretching mode of the α-phase in addition to the two peaks of β-phase at 570 and 631 cm^–1^. This result provides evidence for the presence of SFs from the perspective of the local structure.

Comparison of the Raman spectra reveals remarkable differences between NMCO and NMO, such as overall peak broadening and the emergence of a new peak at 589 cm^–1^. These changes were likely influenced by the diversification of the local oxidation states due to the introduction of Cu^2+^ and Mn^4+^ doping. The JT-active Mn^3+^ and Cu^2+^ as well as non-JT-active Mn^4+^ are expected to show different local symmetries and oxygen bonding strengths, which can contribute to the observed peak broadening. The new peak at 589 cm^–1^ may be attributed to the SF-free nature of the material or to the possible local ordering of Cu and Mn atoms, as suggested in a previous study.^20^ The spectrum of NMZO exhibits three distinct peaks at 568, 588, and 629 cm^–1^, suggesting the presence of an intermediate phase that incorporates structural features of both NMO and NMCO. These changes can be explained by the partial substitution of the Jahn–Teller-active Mn^3+^ with Cu^2+^ or non-active Zn^2+^, which effectively modulates the formation and distribution of the SFs and the *BAB* phase. Thus, Raman spectroscopy confirmed the phases identified by the SXRD and STEM studies (Figure 3f). In addition, Raman analyses provide insights into the local lattice distortions and defects.

To summarize, through synthesis optimization and structural analyses using SXRD, STEM, Raman spectroscopy, and DFT calculations, we elucidated the structural differences among the modulated β-phases. In particular, a crystalline *BAB* phase with an ordered SF distribution was identified for the first time. The electrochemical behaviors of these phases are discussed below.

### Electrochemical Reaction of the NMO Materials

To investigate the impact of divalent cation substitution and different SF structures on electrode performance in SIB cells, we examined the galvanostatic charge–discharge behavior and cycle stability of the three synthesized samples. This comparison provides insight into how the structural differences associated with the SF distribution modulate the redox behavior and structural reversibility during repeated Na extraction and insertion.

Figure 5a shows the initial charge/discharge curves of the cells based on NMO, NMCO, and NMZO. All cells exhibit similar initial discharge capacities of approximately 170 mAh g^–1^. The Coulombic efficiency (CE) of the NMO, NMCO, and NMZO cells were 79.8%, 81.7%, and 83.7%, respectively. The variation in the initial CE can be ascribed to differences in the reactivity toward the electrolyte and the formation of the cathode–electrolyte interphase (CEI). The right of Figure 5a shows the d*Q* d*V* ^–1^ curves for each cell during the initial cycle. The pronounced reversible plateau observed at ∼3.6 V during charge and at ∼3.3 V during discharge is due to the unique slab gliding of the (Mn, Cu)­O_2_ layer.^20^ The corrugated-slab gliding of the β-type structure is perturbed by SFs, and thus the cells with NMO and NMZO that contain SFs do not show the distinct plateau in the voltage range between 3.0 and 3.5 V upon discharging. The NMO cell exhibits multiple voltage steps during charging that are likely associated with structural distortions analogous to those observed in α-NaMnO_2_.^36^ Compared to the NMO and NMCO cells, NMZO displays smoother charge–discharge curves, which is a characteristic often indicative of successful dopant incorporation into the host structure.^21^ The pronounced voltage hysteresis in NMCO originates from sluggish phase-transition kinetics associated with MnO_2_ slab gliding,^20^ which is further impeded by SFs, as evidenced by even higher hysteresis in NMO and NMZO. The structural changes underlying this behavior are discussed in operando XRD results (Figure 5e–g). Figure 5b shows the capacity retention and CE over 50 cycles. The charge–discharge curves are summarized in Figure S13. The NMO cell suffers from a relatively rapid capacity loss. In addition, the scatter of the CE values between the 21st and 27th cycles is likely due to the microshort circuits arising from Mn dissolution.^44,45^ Nevertheless, all cells exhibited superior cycling performance compared to α-NaMnO_2_,^36,46^ suggesting that the zigzag-layered β-phase possesses higher structural reversibility than the planar-layered α-phase. Both Cu and Zn substitutions effectively enhanced capacity retention. The evolution of the CE is in agreement with this trend. The CE of the NMCO cell is stabilized within 10 cycles and reaches >99%, which is sufficiently high in the half-cell with Na metal as a counter electrode without using an electrolyte additive.^47^ This indicates the absence of significant side reactions during cycling. Even at a relatively high upper cutoff voltage of 4.5 V, the NMZO cell also shows a distinct improvement in the capacity retention and the CE compared to the NMO cell, suggesting that Zn doping effectively mitigates Mn dissolution. This trend is likely associated with the structural modifications discussed in the following section. Direct quantification of Mn dissolution is currently in progress and will be presented in future work.

**Figure 5:**
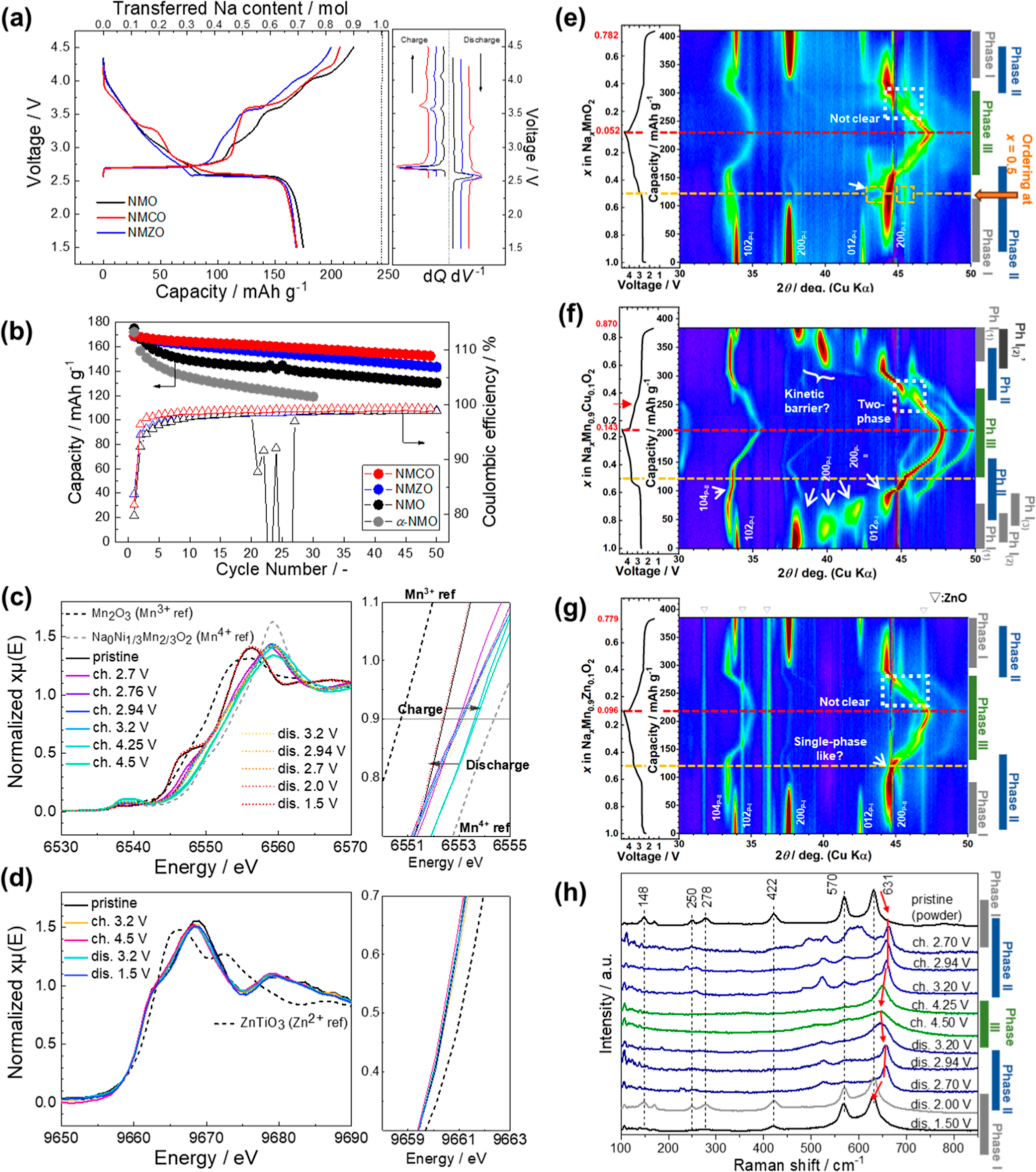
(a) Initial charge/discharge curves of NMO, NMCO, and NMZO cells at a rate of C/20 (≈12 mA g^–1^) with d*Q* d*V* ^–1^ curves (right). (b) Cycling performances and Coulombic efficiency of NMO, NMCO, and NMZO cells. XANES spectra for NMZO of (c) Mn K-edge and (d) Zn K-edge. Heat maps of operando XRD patterns of (e) NMO, (f) NMCO, and (g) NMZO during the first cycle. (h) Ex situ Raman spectra of NMO during the first cycle.

Although both Cu and Zn doping improved the cycle stability, the degree of the improvement and its underlying mechanism are believed to be different for NMCO and NMZO, owing to their different structural characteristics. We further discuss these issues in the next section based on ex situ XRD and Raman characterizations. To evaluate the impact of SFs on Na^+^ diffusivity, rate capability tests were conducted for all three materials (Figure S14). The results show that the effect of SF amount and distribution on rate capability is negligible, indicating that in-plane Na^+^ diffusion is not significantly influenced by SFs. This finding is consistent with previous reports,^20^ which also concluded that the presence of SFs does not disturb.

For all three cells, the redox reactions were investigated using X-ray absorption measurements (Figures 5c,d and S15). Figure 5c shows the Mn K-edge spectra of NMZO and the standard references for Mn^3+^ and Mn^4+^, which were obtained from Mn_2_O_3_ and a desodiated P2-Na_2/3_Ni_1/3_Mn_2/3_O_2_ electrode, respectively. Upon charging, the Mn K-edge exhibited an upshift, indicating the oxidation of Mn^3+^ to Mn^4+^. Upon discharge, the Mn K-edge reversibly shifted back to its original position. By contrast, the Zn K-edge barely changed during charging and discharging. (Figure 5d). This indicates that the Mn­(3+/4+) redox reaction predominantly governs the overall electrochemical process. Although neither Cu nor Zn ions were actively involved in the redox reactions, the Cu spectra exhibited more pronounced changes in the shoulder features (Figure S15c) in contrast to the clear upshift of Cu K-edge observed in NaMn_2/3_Cu_1/3_O_2_.^48^ This behavior reflects the high sensitivity of Cu to variations in oxygen coordination and lattice distortion associated with Mn-oxidation. Similar sensitivity has been reported in the literature for β-Na­(Mn,Cu)­O_2_ and other compounds containing CuO_6_ octahedra.^20^ Because K-edge XANES has limited sensitivity, subtle changes in Cu-oxidation state cannot be excluded. Future investigations employing more sensitive techniques, such as EXAFS fitting and Cu L-edge XAS, will be crucial to elucidate Cu’s redox behavior.

### Structural Evolution during First Na Extraction and Insertion

To investigate the effect of the structural differences induced by divalent cation substitution on the electrochemical activity and cycle life, operando and ex-situ XRD measurements were performed. Figure 5e–g show heat maps of the operando XRD patterns collected during the first charge–discharge cycle for NMO, NMCO, and NMZO cells, respectively. All three samples show three distinct regions corresponding to the charge curves, (i) the long plateau below 3.0 V, (ii) the potential jump from 2.7 to 3.5 V and (iii) the slope above 3.5 V. Upon discharge, the trend is almost reversible except for the second region and only NMCO exhibited a reversible potential jump. At the end of the discharge, all diffraction peaks returned to their original positions, indicating reversible structural changes. Our group recently revealed the phase evolution of 12.5% Cu-doped β-NaMnO_2_, demonstrating three phases: Phase I is the β-phase, including the as-prepared state; Phase II is characterized by the gliding of MnO_2_ corrugated layers along the *b*-axis; and Phase III involves slab gliding along the *a*- and *b*-axes. The three regions can be assigned to the following reactions: (i) a two-phase reaction between Phases I and II, (ii) a single-phase reaction of Phase II, and (iii) a single-phase reaction of Phase III.^20^ Thus, we can assign reversible phase transitions based on the initial structures prior to Na extraction.

Slab gliding along the *a*-axis is essential for the formation of Phase III; hence, SFs along this direction play a critical role. NMCO exhibited a distinct evolution of Phase III with high crystallinity, which can be attributed to its nearly SF-free structure.^20^ By contrast, both NMO and NMZO showed significantly broader diffraction peaks associated with Phase III, indicating that the presence of SFs severely hindered slab gliding. Notably, NMO displays even broader peaks than NMZO, suggesting that the *BAB* superstructure obstructs slab gliding more effectively than the randomly distributed SFs. This difference in the spatial distribution of the SFs between NMO and NMZO plays a crucial role in governing the overall phase evolution, in addition to influencing the formation of Phase III. As a result, NMZO demonstrated a clearer separation between Phase I and Phase II and a more highly crystalline Phase II compared to NMO. Because the α-type domain separates the β-domains within the *BAB* superstructure, the intrinsic phase transitions of the β-phase may be blocked. This structural constraint leads to NMO exhibiting less distinct phase transitions, as evidenced by the broader diffraction peaks. By contrast, in NMZO, the randomly distributed SFs can decouple the structural changes at the slip planes; however, the domains between the slip planes undergo typical phase evolution. A structural anomaly is observed only in NMO at the sodium extraction level of approximately 0.5 mol, as indicated by the orange arrow and squares in Figure 5e. This anomaly is likely due to the long-range ordering of sodium ions and vacancies, as well as the charge ordering between Mn^3+^ and Mn^4+^ ions presumably within the *BAB* domains, as reported in O′3-NaMnO_2_ ^5^. Those differences in structural evolution between NMO and NMZO help explain why the initial charge–discharge curves (Figure 5a) cannot be attributed solely to the number of SFs. Instead, the distribution of SFs likely plays a critical role in determining the electrochemical behavior.

In NMCO, Phase I can be further subdivided into three distinct stages (I(1)–I(3)) during charging, indicating the presence of metastable β-phases with different Na concentrations while retaining the structure with the *Pmmn* space group. The large differences in the *a*-axis length among these stages indicate that the stabilization energy of the Jahn–Teller distortion plays an important role in their formation. However, upon discharge, Phase I(3) is not observed, and the 200 reflection at approximately 40° exhibits a significantly higher intensity. This asymmetric structural change was further verified by comparing first discharge and second charge (Figure S16a,b). During the first cycle, no significant structural degradation such as irreversible phase transformation was observed. Although the operando cell introduces a considerable background signal at low angles (2 theta <25°), the in situ XRD results confirm that there is no trace of the 001_*a*_ diffraction peak (Figure S16a–c). This observation is consistent with ex-situ XRD and Raman analyses after one cycle (Figures 6 and S17), where we further discuss the prolonged structural changes up to 50 cycles. The gradual emergence of the *a*-phase becomes evident only after extended cycling, as shown in the XRD patterns after approximately 10 cycles.

**Figure 6:**
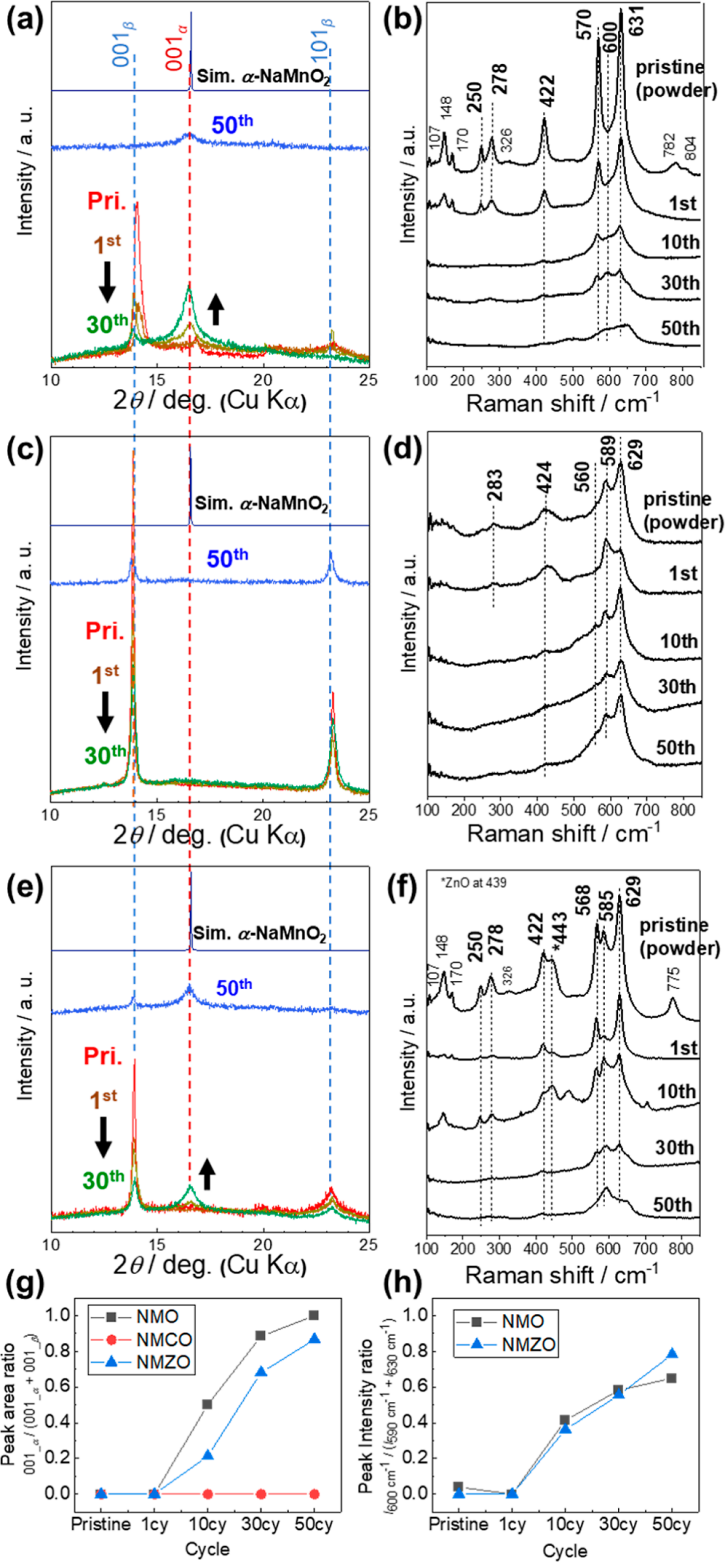
Structural degradation of NaMn_0.9_Me_0.1_O_2_ (Me = Mn, Cu, Zn). Ex-situ XRD (left) and Raman (right) results for electrodes after 1, 10, 30, and 50 cycles for (a,b) NMO, (c,d) NMCO, and (e,f) NMZO cells. Each electrode was kept at 2.0 V. (g) *α/β* phase ratio based on the area of 001 diffraction peak. (h) α/β phase ratio based on the peak intensity of the Raman peaks at 600 and 630 cm^-1^.

Figure 5h shows the ex-situ Raman spectra collected at various charge states and discharge depths during the first cycle of NMO. The spectra exhibit high reversibility, as evidenced by the close match between the fully discharged and pristine states. To the best of our knowledge, this work is the first detailed Raman analysis of ex-situ electrodes in the Na_*x*_MnO_2_ material family, most likely because such analysis is challenging due to the sensitivity of the electrodes to laser-induced damage. Here, we focus on the evolution of the A_g_-like peak at 631 cm^–1^ during the first cycle. As the overall spectral changes are considerably complex, they will be addressed in future work. The peak upshift to ∼650 cm^–1^ observed immediately after desodiation coincides with the appearance of Phase II with a shorter *a*-axis length, which is attributed to the strengthening of the Mn–O bond through the oxidation of trivalent manganese to its tetravalent form.^39^ In Phase III, the peak is significantly broadened, which may be due to either structural amorphization or measurement artifacts because beam damage tends to be more pronounced for the charged electrodes.^49^ This trend is highly reversible upon discharge. Thus, the complementary use of XRD and Raman spectroscopy provides a powerful framework for understanding both average and local structures, particularly in materials exhibiting significant lattice defects and distortions, such as the present system.

### Degradation Mechanisms of Electrochemical Performance

To understand the impact of divalent cation substitution on capacity degradation, we performed postcycling evaluations using XRD, Raman spectroscopy, SEM, and XAFS analyses.

Figures 6a,c,e (magnified) and S17 (entire 2θ range) show the ex situ XRD patterns of pristine NMO, NMCO, and NMZO, as well as those collected after 1, 10, 30, and 50 charge-discharge cycles. For all samples, the intensity of the 001_β_ diffraction peak located near 14° that originates from the zigzag-layered β-phase gradually decreased and became broader with increasing cycle number. In particular, this peak was no longer detectable in NMO after 50 cycles. Additionally, in both NMO and NMZO, the intensity of the 001_α_ diffraction peak associated with the α-phase increased with cycling. By contrast, the 001_α_ peak was not observed at any stage in NMCO.

Focusing on the 101_β_ reflection near 23.3°, which is an indicator of the number of SFs, it is observed that NMCO maintained the distinct diffraction peak even after 50 cycles. For NMO, the peak disappeared after 30 cycles, whereas in NMZO, the initially weak peak exhibited a slight decrease in the intensity. The SEM images of the electrodes after the first and 50th cycles are shown in Figure S18. After the first cycle, all samples exhibited minimal particle cracking. However, after 50 cycles, numerous cracks were observed in NMO and NMZO, whereas NMCO showed significant suppression of crack formation. These cracks are likely due to interlayer delamination along the *c*-axis. Ex-situ XANES measurements (Figure S19) also revealed an increase in the oxidation state of Mn during cycling for NMO, which is consistent with capacity degradation. The ex-situ XRD results indicate that α-phase formation during cycling is slow in the SF-free β-domain as confirmed for NMCO because its growth is initiated selectively at SFs. Notably, the presence of the *BAB* superstructure appears to accelerate the formation of the undesired α-phase.

A deeper understanding of how these initial structures influence the tendency toward unfavorable structural transition can be obtained by integrating these findings with the operando XRD results (Figure 5e,f). In our previous work, we reported that Cu-doped samples without SFs undergo slab sliding upon Na extraction without introducing significant lattice strain.^20^ As illustrated schematically in Figure S20, this slab sliding occurs smoothly without generating lattice strain when SFs are absent. In contrast, the presence of SFs suppresses slab gliding, leading instead to localized lattice distortion, which triggers the *a*-phase transition. For *BAB*-type structures, pre-existing ordered planar domains oriented selectively in one direction accelerate this transition under strain, resulting in more pronounced structural degradation. Therefore, we conclude that the presence of SFs, particularly ordered SF distributions such as those in *BAB* structures, significantly promotes structural changes and thus negatively impacts cycling stability.

Figure 6b,d, and f show the ex-situ Raman spectra of NMO, NMCO, and NMZO collected from the pristine samples and the samples after 1, 10, 30, and 50 cycles. For NMO, the Raman peaks observed in the pristine state broadened significantly with increasing number of cycles, indicating a pronounced loss in crystallinity during cycling. By contrast, NMCO retained its peaks at 560, 589, and 629 cm^–1^ even after 50 cycles, suggesting that the crystal structure remained relatively intact. NMZO also retained a peak near 585 cm^–1^ after 50 cycles, indicating that although its structure was not as robust as that of NMCO, it retained higher crystallinity than NMO after prolonged cycling.

Figure 6g presents the evolution of the phase ratios during cycling derived from the integrated peak areas of the 001_β_ and 001_α_ diffraction peaks for NMO, NMCO, and NMZO (Figure 6a,c and e). A pronounced phase transition from the β-phase to the α-phase was observed after 10 cycles, indicating structural instability compared to NMCO, with no trace of the 001_α_ signal. Notably, Zn doping effectively decreased the rate of this transformation, suggesting that it plays an important role in suppressing the growth of α domains during repeated Na insertion/extraction. Figure 6h shows the evolution of the Raman peak ratios assigned to the β- and α-phases. Here, the peaks at approximately 631 cm^–1^ and 600 cm^–1^ represent the β- and α-phases, respectively. Peak fitting of the Raman spectra was conducted, and good fitting quality was obtained (Figure S21). The evolution of the peak ratio closely matched the phase ratio calculated from the XRD data shown in Figure 6g, thereby cross validating the Raman peak assignment. Notably, the formation rate of the α-phase appears to be unaffected by Zn-substitution in the Raman analysis. This suggests that while the rate of local formation of SFs remains unchanged, their growth into extended α-phase long-range crystalline domains is effectively suppressed by the random distribution of Zn. After the β-phase disappears, structural convergence likely leads to similar degradation trends between NMO and NMZO, as indicated by the comparable degradation slope observed after 30 cycles (Figure 5b), even though the disappearance occurs at different cycle numbers. Notably, pure α-NaMnO_2_ exhibits much poorer cycling stability even under lower cutoff voltages,^36^ suggesting that the initial β-like rod-shaped morphology plays a beneficial role in mitigating degradation during long-term cycling. The superior cycling stability of the α-phase transformed from the β-phase calls for further investigation. These findings underscore the role of Zn doping in modulating the domain connectivity rather than local SF nucleation. The combined analytical techniques demonstrate the significant structural enhancement obtained by the absence of SFs achieved through the Cu doping, compared to the non-doped β-phase. Interestingly, Zn doping also improves cycle life, even though it does not suppress SF formation.

These results suggest that the presence of a large *BAB*-type superstructure domain is detrimental to cycle life, and Zn doping effectively inhibits the growth of such domains.

## Conclusions

In this study, we synthesized pristine, Cu- and Zn-substituted β-NaMnO_2_ samples and investigated the effects of SF-distribution on their crystal structures and electrochemical properties. Careful synthesis optimization and STEM analysis revealed that each material exhibited distinct structural features. In particular, the undoped NMO displayed a *BAB*-type superstructure, characterized by the presence of α-domains sandwiched between β-domains. The key difference between Cu and Zn substitutions lies in their effects on SFs and superstructure formation. While Cu doping results in an SF-free β-phase, Zn doping maintains a similar degree of SFs as that in the non-doped NMO, while effectively suppressing the formation of the *BAB*-type superstructure.

All three samples, NMO, NMCO, and NMZO, exhibited highly reversible electrochemical activities with an initial discharge capacity of approximately 170 mAh g^–1^. Combined operando XRD and ex-situ Raman spectroscopy analyses established a correlation between the phase transitions and microscopic phonon modes for the first time. The capacity retention strongly depends on the intrinsic structural features of each sample, and in particular on the SF distribution. In NMO, SFs are arranged in an ordered *BAB*-type superstructure. This structural configuration leads to rapid capacity decay, which is attributed to the undesired formation of α-domains triggered by the *BAB* domains. By contrast, the random distribution of SFs in NMZO did not negatively affect the cycle life. The phase changes over repeated cycles were validated by ex-situ XRD and Raman spectroscopy analyses.

Given that defect chemistry, represented in this study as planar defects within the *bc*-plane, is a critical and often unavoidable aspect in the synthesis of crystalline materials, and particularly layered materials, the elucidation of its influence on electrochemical reversibility provides a new perspective for the rational design of durable NIBs. These findings can be extended to other layered oxide systems beyond NIBs such as Li-ion batteries and other post-Li-ion batteries. Furthermore, this study provides fundamental insights into solid-state chemistry of Na_*x*_MnO_2_ materials by revealing how such defects evolve dynamically during Na extraction and insertion, thereby bridging the gap between atomic-scale structural imperfections and macroscopic electrochemical performance.

## Supplementary Material



## Notes

Experimental section for material synthesis, material characterization (XRD, SEM, TEM, XAS, Raman measurements), electrochemical measurements, and DFT calculations; SEM/EDS images; summary table of lattice constants; XRD patterns in various gas flow; SAED patterns; STEM/EDS mapping; structural parameter of *BAB* structural model; STEM images of *a-c* plane slippage; STEM images along the [100] axis; DFT calculations; phonon density of states; simulation of Raman modes; electrochemical curves; XANES spectra; operando XRD profiles; ex-situ XRD patterns; ex-situ SEM images; ex-situ XANES spectra; fitting results of ex-situ Raman spectra (PDF)

## ⊥

Ryoichi Tatara, Department of Chemistry and Life Science, Yokohama National University, Yokohama, 240–8501, Japan
