# Supplementary material for: Defect Electrochemistry in Stabilizing Corrugated Layered NaMnO2
Source: J Am Chem Soc. 2026 Jan 29;148(11):11654–66. doi: 10.1021/jacs.5c19128 (PMC13022877; doi:10.1021/jacs.5c19128)
Supplement: Supplementary file 1 [file ja5c19128_si_001.pdf]

Supporting Information

Defect Electrochemistry in Stabilizing Corrugated Layered $NaMnO_2$

Shinichi Kumakura,*[,#,ζ,]* Yusuke Miura,[#,ζ] Kei Kubota,[†] Ryoichi Tatara,[ζ,##] Eun Jeong Kim,[ζ] Huu Duc Luong,[ψ] Yoshitaka Tateyama,[†,ψ] Yoshinobu Miyazaki,[‡] Tomohiro Saito,[‡] and Shinichi Komaba[ζ,]*

[ζ] Department of Applied Chemistry, Tokyo University of Science, Shinjuku, Tokyo, 162-8601, Japan

[†] Research Center for Energy and Environmental Materials (GREEN), National Institute for Materials Science, 1-1 Namiki, Tsukuba, Ibaraki 305-0044, Japan

[ψ] Institute of Integrated Research (IIR), Institute of Science Tokyo, Midori, Yokohama, Kanagawa 226-8501, Japan

[‡] Tsukuba Satellite Laboratory, Sumika Chemical Analysis Service (SCAS), Ltd., Tsukuba, Ibaraki 300-3266, Japan

[#] S.Kumakura and Y.Miura contributed equally to this work.

[##] *Ryoichi Tatara, Department of Chemistry and Life Science, Yokohama National University, Yokohama, 240-8501, Japan*

Keywords: Sodium-ion batteries, Stacking faults, Sodium manganese oxide, Defect engineering, Phase transition

*Corresponding author

E-mail:komaba@rs.tus.ac.jp

kumakura@rs.tus.ac.jp

Experimental

Material synthesis

Layered oxide samples $NaMnO_2$, $NaMn_{0.9}Cu_{0.1}O_2$ and $NaMn_{0.9}Zn_{0.1}O_2$ (hereafter, NMO, NMCO, and NMZO, respectively) were synthesized through high-temperature solid-state reactions. Stoichiometric Na_2CO_3 (Nacalai Tesque, purity >99.8%), CuO (Kanto Chemical, purity >98.0%), ZnO (Kanto Chemical, purity >99.5%), and Mn_2O_3 prepared by calcining $MnCO_3$ (Sigma-Aldrich, purity > 99.9%) at 700 °C in air for 5 h, were employed as starting materials. Excess sodium (8 mol%) in the form of Na_2CO_3 was added for the synthesis of NMO and NMZO to compensate for sodium evaporation during firing. These reagents were mixed via ball milling with acetone at 600 rpm for 12 h using a planetary ball mill (PULVERISETTE 7 classic line, Fritsch). The mixtures were then dried and pressed into pellets. Each pellet was heated to 1050 °C at a heating rate of 1 °C/min in air for NMCO and NMZO, and in oxygen flow for NMO. Upon reaching 1050 °C, the samples were immediately transferred to an Ar-filled glovebox, cooled to room temperature in the glovebox and kept inside to avoid contact with the moisture in the air. For NMO and NMZO, the heated pellets were ground with a pestle and mortar, re-pelletized, and reheated under the same operating conditions. This procedure was repeated four times; thus, five calcination cycles were performed for NMO and NMZO and one calcination cycle for NMCO. The samples used as references for the Raman spectra were synthesized following the procedures used in previous studies on α-$NaMnO_2$[1] and orthorhombic $LiMnO_2$[2].

Material characterization

X-ray diffraction (XRD)

Conventional powder X-ray diffraction (XRD) measurements were performed using a SmartLab instrument (Rigaku) equipped with a high-speed 1-dimensional detector (D/teX Ultra, Rigaku) using Ni-filtered Cu Kα radiation in Bragg–Brentano geometry. For *ex situ* XRD measurements, the electrodes cycled in the coin cells (described below) were removed from the cells. The electrodes were rinsed with propylene carbonate (PC) and diethyl carbonate (DEC) to remove the electrolyte and degradation products; they were then dried at room temperature in an Ar-filled glovebox. A custom-built airtight holder was used for XRD measurements to avoid exposure to air.

Synchrotron XRD (SXRD) measurements were conducted at BL02B2 in SPring-8 in Japan using a wavelength of 0.50015 Å calibrated with NIST SRM 674b CeO_2. Powder specimens were placed in a Lindemann glass capillary (φ 0.3 mm) inside an Ar-filled glovebox and sealed by melting the capillary end to prevent sample degradation by air moisture. Diffraction patterns were collected using a large Debye–Scherrer camera.[3]

A two-electrode *operando* cell (Rigaku) with a beryllium window was employed for *operando* XRD measurements. The diffraction patterns were collected using a MultiFlex instrument (Rigaku) equipped with a high-speed 1-dimensional detector (D/teX Ultra, Rigaku) using Ni-filtered Cu Kα radiation in Bragg–Brentano geometry. Tracking of the structural evolution was facilitated by drawing *operando* XRD heat maps. Owing to scattering from the *operando* cell and separators, the intensity of each pattern at a lower angle was extremely

high. Therefore, each baseline was subtracted along the fitted (B-)spline curve. In addition, undesirable peaks due to the cell components, such as the Be window and Al foil, were subtracted as fixed values. Rietveld refinement was performed using RIETAN-FP.[4] Crystal structures were drawn using VESTA.[5]

Morphological and Chemical Characterization

Scanning electron microscopy (SEM) was performed using a JCM-6000 (JEOL) instrument equipped with a thermionic emission gun at an acceleration voltage of 15 kV. SEM-EDS (energy dispersive X-ray spectroscopy) spectra were obtained with a JED-2200 detector (JEOL).

Transmission electron microscopy (TEM) observations were carried out with a JEM-ARM200F instrument as follows. The specimens for observation along the [100] zone axis were directly dispersed onto TEM grids in an Ar-filled glovebox and transferred to the microscope using a vacuum transfer holder. The specimens for observation along the [010] zone axis were processed using cryo-focused ion beam (cryo-FIB) milling with gallium ions to fabricate thin samples. Then, the specimens were transferred to the microscope without exposure to the air via an Ar-filled glove box.

Atomic-resolution STEM images were acquired using a JEM-ARM200F instrument (JEOL) with a CESCOR (CEOS) spherical aberration corrector (Cs corrector) operated at 200 kV according to the report by Masese et al.[6] The specimens were pretreated as described above for the TEM measurements. The probe convergence semi-angle was set to 20 mrad and the probe current was maintained at 23 pA to reduce damage to the specimen by electron-beam irradiation. The acceptance semi-angles of the detector for the annular bright-field (ABF) and high-angle annular dark-field (HAADF) were set to 11–23 mrad and 90–370 mrad, respectively. Atomic-resolution STEM images were obtained by accumulating approximately 20 images with an exposure time of 0.5 s per image.[7] The S/N ratio is improved by extracting and averaging approximately eight crystallographically equivalent regions. STEM-EDS spectra were collected with two JED-2300T detectors (JEOL); STEM-EDS mappings were obtained with Noran NSS (Thermo Fisher Scientific).

X-ray absorption spectroscopy (XAS)

X-ray absorption spectroscopy (XAS) measurements were conducted at the BL-12C beamline of the Photon Factory (PF), High Energy Accelerator Research Organization (KEK), Japan. For sample preparation, the pristine powder was first mixed with boron nitride using an agate mortar and pestle, and then pressed into pellets using a 7-mm-diameter die under a load of approximately 1 t for 3 min. *Ex situ* samples were prepared by holding the cell at the target voltage for approximately 24 h, after which the coin cells were disassembled and the electrodes were collected. The electrodes were washed with propylene carbonate (PC) and diethyl carbonate (DEC), and then dried. Subsequently, the composite electrodes were carefully peeled from the current collectors. Both the pelletized pristine and electrode samples were sealed in Kuraray EVAL film to prevent exposure to air. All sample preparation procedures were performed in an argon-filled glovebox to avoid atmospheric contamination.

Raman spectroscopy

Specimens for Raman spectroscopy were prepared in an argon-filled glovebox to prevent atmospheric exposure. Powdered samples or electrodes peeled from the current collectors were placed on glass slides and sealed with a cover glass using vacuum-grade silicone grease (Shin-Etsu Silicone). For the powder samples, care was taken to ensure the formation of a densely packed layer without voids. For both powder and electrode preparations, the sample was gently pressed onto a slide using a clean pair of tweezers. Raman spectroscopy was conducted using a RAMANtouch/RAMAN-11i system (Nanophoton, Bruker) and a Nikon Plan Fluor 60x/0.85) objective lens. The excitation laser had a wavelength of 532.0 nm and output power of approximately 2 mW. The spectrometer settings included a 600 gr/nm diffraction grating, a slit width of 50 µm, and an acquisition time of 200 s per scan, accumulated over three scans. Prior to all measurements, the spectrometer was calibrated using a silicon substrate. The acquired spectra were linearly shifted such that the main Si peak appeared at 520.7 cm^{-1}.

Electrochemical measurements

A slurry was prepared by mixing the pristine active material, acetylene black (AB, Strem Chemicals), and polyvinylidene difluoride (PVdF, Polysciences) in a weight ratio of 80:10:10, followed by dispersion in *N*-methyl-2-pyrrolidone (NMP, Kanto Chemical) using a mortar and pestle. Positive electrodes were prepared by casting the slurry on aluminum foil and drying it overnight at 80–110 °C under vacuum. The electrodes were punched into discs of diameter either 10 or 15 mm with a typical active material loading of 2 mg cm^{-2}. The electrodes were pressed between titanium foils at a pressure of 1 t for 3 min. For the coin-cell test, an R2032 type cell with an Al-clad stainless-steel cap (Hosen Corp.) was used. A glass fiber filter (GB-100R, ADVANTEC) was used as the separator and 1.0 mol dm^{-3} $NaPF_6$ dissolved in propylene carbonate (PC, Kishida Chemical) was used as the electrolyte solution. The negative electrode was sodium metal foil (Kanto Chemical, purity >99%). The two-electrode *operando* cell (Rigaku) was constructed using the same procedure as that described earlier for the coin cell with two differences: the slurry was cast on thin (~10 µm) aluminum foil (Nilaco), and electrodes were cut into pieces of diameter 15 mm. The cut electrodes were pressed between titanium foils at a pressures of 0.5 tons for 3 min and two separators were placed between the electrodes. All procedures were conducted in an Ar-filled glove box.

Galvanostatic charge–discharge tests were carried out using a TOSCAT-3100 instrument (TOYO System) maintained at 25 °C. The current density was set to C/20 (≈12 mA/g). For *ex situ* XRD measurements, the constant current/constant voltage (CC/CV) mode was adopted, after which the target voltage (2.0 V) was maintained for at least 20 h to achieve an equilibrium state. For the operando analysis, an SP-200 instrument (Bio-Logic Science Instruments) was used for charge and discharge, and the current density was set to C/40 (≈ 6 mA/g).

DFT Calculation

All spin-polarized DFT energy calculations were implemented employing Vienna *Ab initio* Simulation Package (VASP) with the projector augmented wave (PAW) pseudo-potentials.[8,9] Perdew–Burke–Ernzerhof (PBE) generalized gradient approximation (GGA),[10] adding Hubbard-like (GGA+U method) terms were applied: U_{Mn}=

4.0 eV[11,12] following the Dudarev scheme[13] to overcome the overestimation of 3d orbitals delocalization in transition metals. Additionally, the DFT-D3 functional[14] was employed to include van der Waals correction term, as this functional provides better agreement with experimental results.[15,16] The cut-off energy of 520eV and the 10×10×5, 6×9×4 and 9×5×4 k-point meshes were utilized for the α-$NaMnO_2$, β-$NaMnO_2$, and BAB $NaMnO_2$ phases, respectively. The primitive cells of $NaMnO_2$ at different phases were fully optimized until the residual forces on atoms are smaller than 5×10^{-3} eV/atom Harmonic phonon calculations were performed using the phonopy code[14,17] to identify the vibration frequencies and mode eigenvector at the Γ points. Force-constant matrices for the α-$NaMnO_2$, β-$NaMnO_2$ and BAB $NaMnO_2$ phases were computed using 3×3×2, 2×3×2 and 3×2×2 supercells, containing 72, 96, and 144 atoms, respectively. The phonon spectrum indicates that the considered structures are stable. The Raman activities were obtained using the Phonopy-Spectroscopy script.[18]

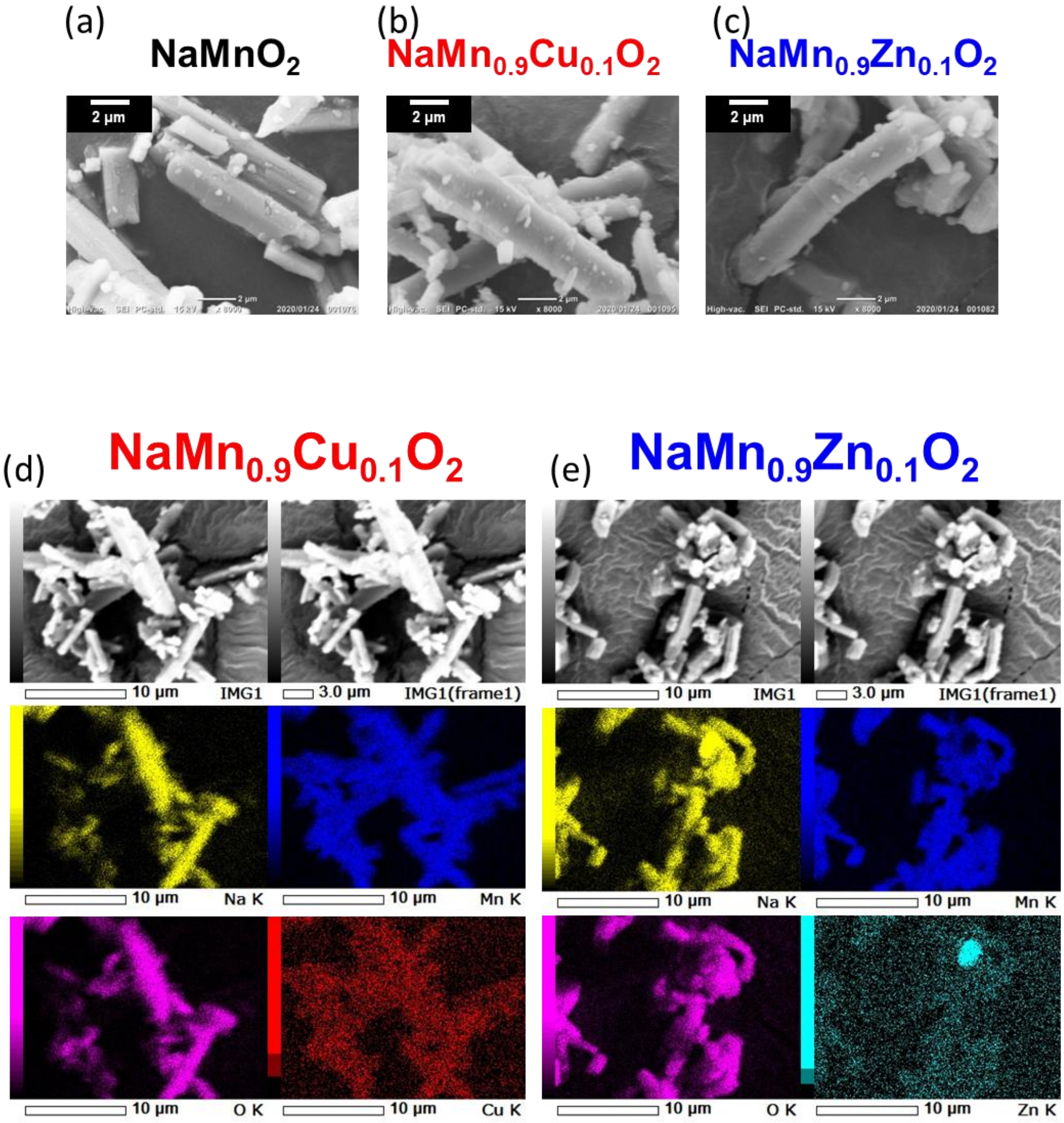

Figure S1. (a-c) SEM images and (d,e) EDS mapping of $NaMn_{0.9}Me_{0.1}O_2$ (Me = Mn, Cu, Zn).

Table S1. Lattice constants of *β*-$NaMn_{0.9}Me_{0.1}O_2$ (Me=Mn, Cu, Zn)

Me	*a* (Å)	*b* (Å)	*c* (Å)
Mn	4.7830(1)	2.8567(1)	6.3276(1)
Cu	4.7549(1)	2.8586(1)	6.3289(1)
Zn	4.7765(1)	2.8581(1)	6.3303(2)

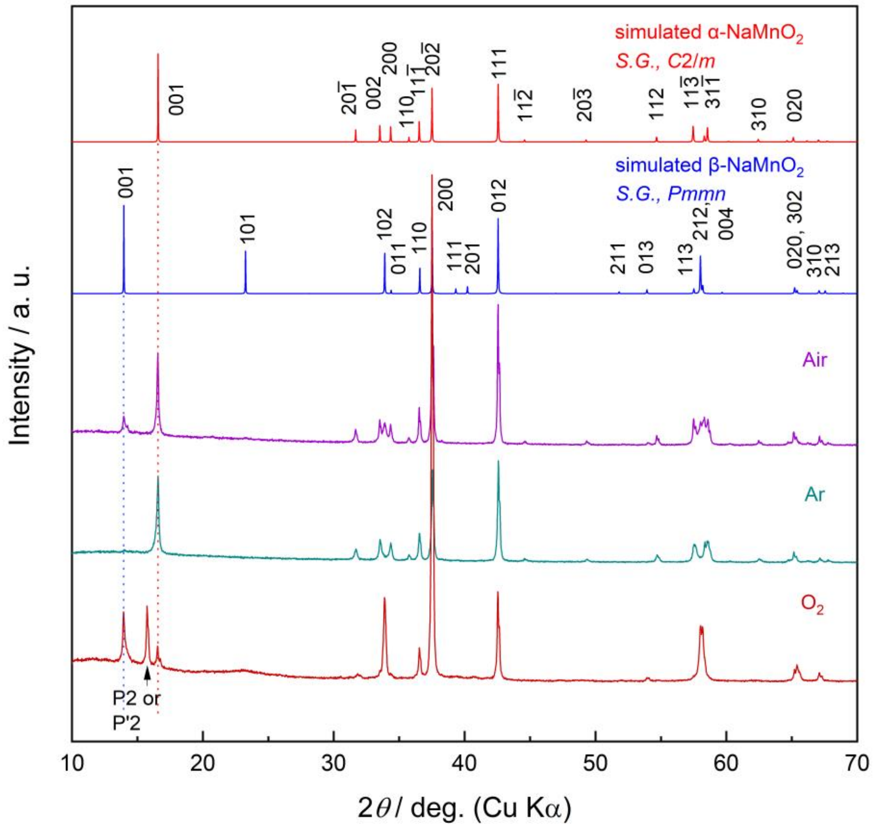

Figure S2. XRD patterns of $NaMnO_2$ fired under air, argon, and oxygen. No excess of sodium was added to the starting material. Each pellet was heated to 1050 ºC at a heating rate of 1 ºC/min at 50 mL/min of gas flow, and then quenched. XRD patterns of powders obtained by single firing NMO pellets in air atmosphere in a muffle furnace or in argon flow or oxygen flow in a tube furnace. Interestingly, each atmosphere yielded a different crystal structure. Focusing on the diffractions from 10° to 20°, the air-calcined NMO exhibited a mixed phase of α-$NaMnO_2$ and β-$NaMnO_2$, where the intensity of the 001 reflection from the β-phase at 13.9° was lower than that of the α-phase at 16.5° ($I_{001\beta} < I_{001\alpha}$). In contrast, O_2-calcined NMO showed additional reflections at 15.7°, attributed to the 002 reflection of P2- or P′2-$Na_{2/3}MnO_2$,[19,20] indicating a three-phase mixture of α-phase, β-phase, and P2/P′2-type phases. These P2-type phases, which contain a lower Na content (2/3 mol%) compared to α- or β-$NaMnO_2$, likely formed due to Na volatilization from the pellet surface under the oxidizing O_2 atmosphere. Despite this, the 001 reflection from the β-phase remained stronger than that from the α-phase ($I_{001\beta} > I_{001\alpha}$), suggesting that O_2 calcination favors β-NMO phase formation. However, when calcined under Ar flow, the β-phase 001 reflection was absent, and single-phase α-$NaMnO_2$ was obtained. These results indicate that calcination in an oxygen-rich atmosphere is preferable for achieving single-phase β-NMO.

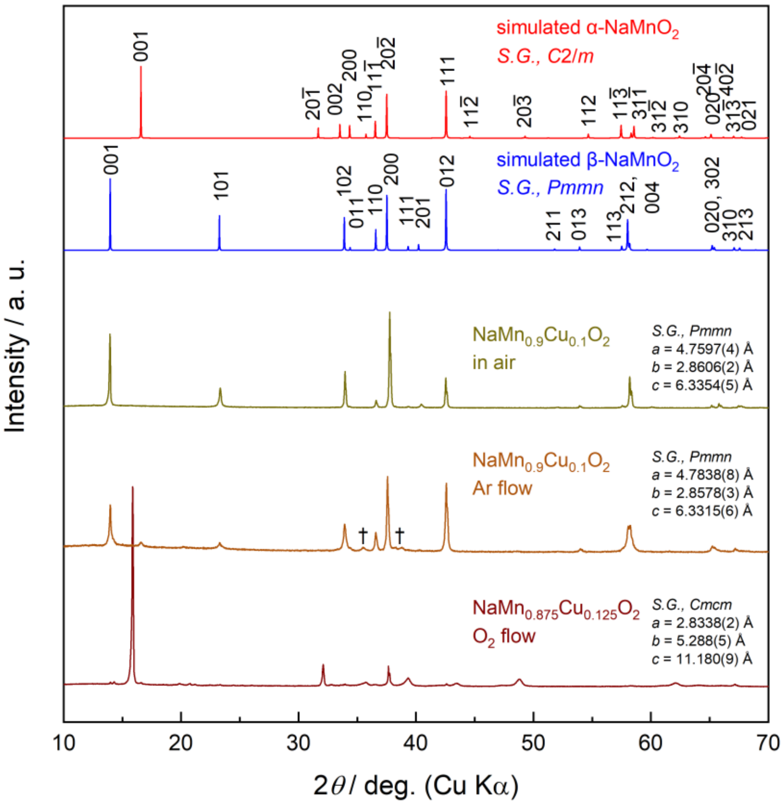

Figure S3. XRD patterns of $Na_{1.05-\delta}Mn_{0.875}Cu_{0.125}O_2$ fired under air, argon, and oxygen. NMCO pellets were calcined under O_2 and Ar flows. Under O_2, the main phase was P′2-type, whereas under Ar, β-phase was still predominant, although poor crystallinity and unreacted CuO were observed. The lack of complete CuO incorporation under Ar is likely due to the relatively reducing nature of Ar compared to air, which suppresses the oxidation of Mn^{3+} to Mn^{4+} necessary for Cu^{2+} to substitute into the β-phase lattice. When an NMCO pellet first calcined in air was reground and recalcined under Ar, the resulting sample showed improved β-NMCO crystallinity compared to those calcined only under Ar. Nevertheless, CuO appeared as a secondary phase, which was not observed after the initial calcination. These results demonstrate that air atmosphere is optimal for obtaining single-phase β-NMCO, whereas O_2 is unsuitable. Furthermore, in the case of NMCO, the stabilizing effect of Cu substitution on the β-phase appears to outweigh the α-phase-favoring effect of Ar calcination.

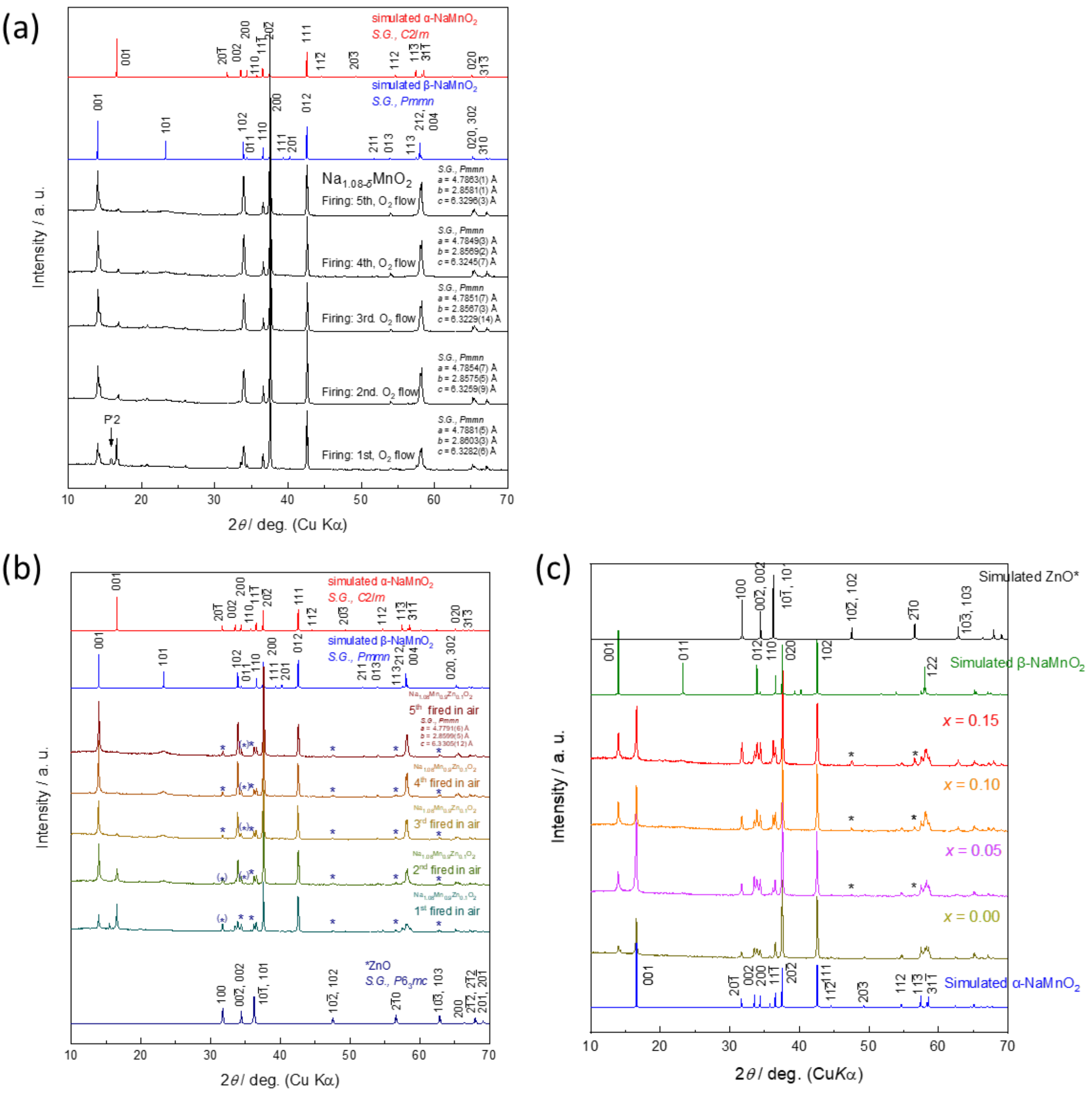

Figure S4. XRD patterns of successive calcination for (a) NMO in O_2 and (b) NMZO in air, (c) XRD patterns of Zn amount series, $NaMn_{1-x}Zn_xO_2$. In air-calcined NMO, the $I_{001\beta}$ / $I_{001\alpha}$ ratio gradually increased with repeated calcination. Comparing the XRD profiles from 1 to 5 calcination steps, the FWHM of the 001_β reflection decreased from 0.22° to 0.12°, and the crystallite size along [001] increased from 370 Å to 725 Å.

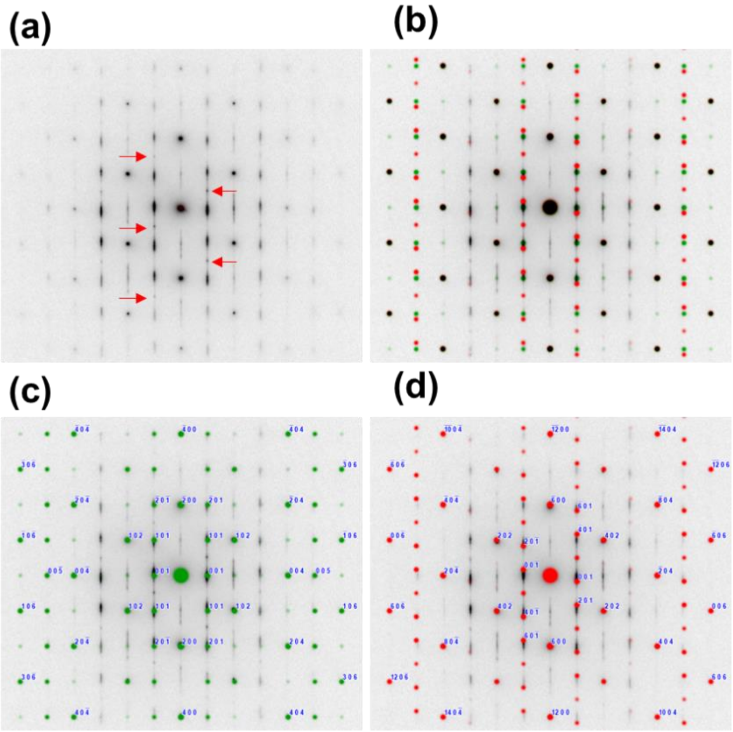

Figure S5. SAED patterns of β-$NaMnO_2$ along the $[010]_\beta$ axis. (a) A Color-inversed SAED pattern (same field as Figure 2a). (b) A Simulated SAED pattern with the experimental one. The diffractions are indexed as orthorhombic β-$NaMnO_2$ lattice (S.G., *Pmmn*) marked as green spots, and modulated monoclinic lattice (S.G., *C*2/*m*) marked as red spots. If the simulated reflection position overlaps, the spot is illustrated as black spots. Each index is shown in (c) orthorhombic β-$NaMnO_2$ and (d) modulated monoclinic *BAB* phase. The red arrowheads point to the diffractions from the modulated monoclinic lattice.

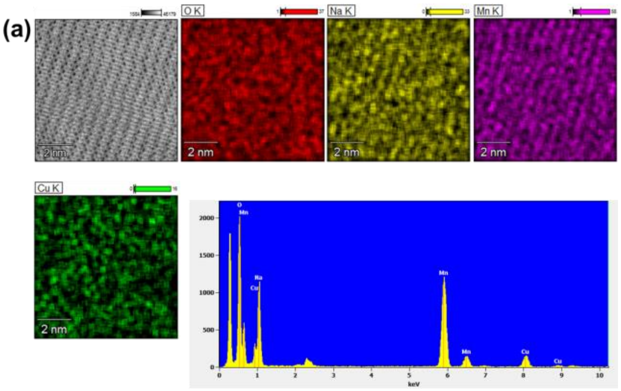

Atom	Counts	Atomic %
O K	8235	38
Na K	8034	30
Mn K	18223	27
Cu K	2831	4.4

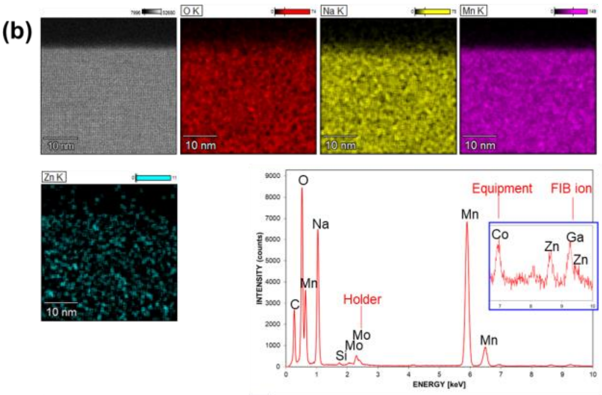

Atom	Counts	Atomic %
O K	38659	34.3
Na K	48224	35.3
Mn K	102853	30.1
Zn K	921	0.29

Figure S6. STEM-EDS analysis for (a) NMCO-2 and (b) NMZO.

Table S2. Structural parameter of the *BAB* super structure

Structural parameters for *BAB* phase				
Space group: *C2/m*<br>a = 14.685 Å, b = 2.850 Å, c = 6.330 Å, α = 100.2°,<br>V = 258.70 Å^3				
Atom	x	y	z	g
Na1	0	0	0	1.0
Na2	0.33333	0	0.16824	1.0
Mn1	0	0	0.5	1.0
Mn2	0.33333	0	0.66504	1.0
O1	0.16667	0	0.12684	1.0
O2	0.16667	0	0.53684	1.0
O3	0.5	0	0.295	1.0

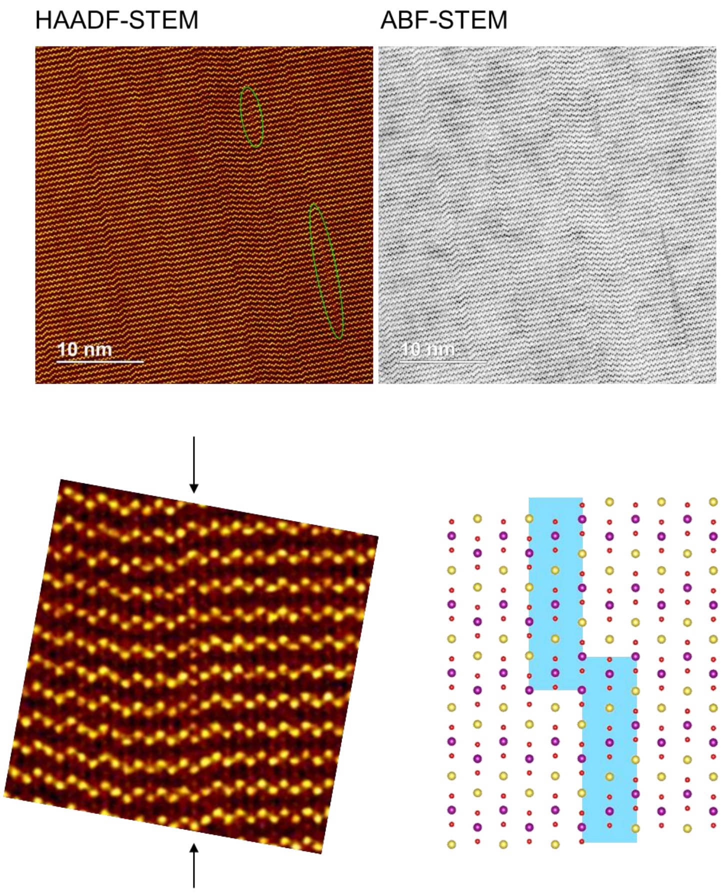

Figure S7. The slippage of twinning plane. STEM images of NMCO-2 along the *a*-*c* plane.

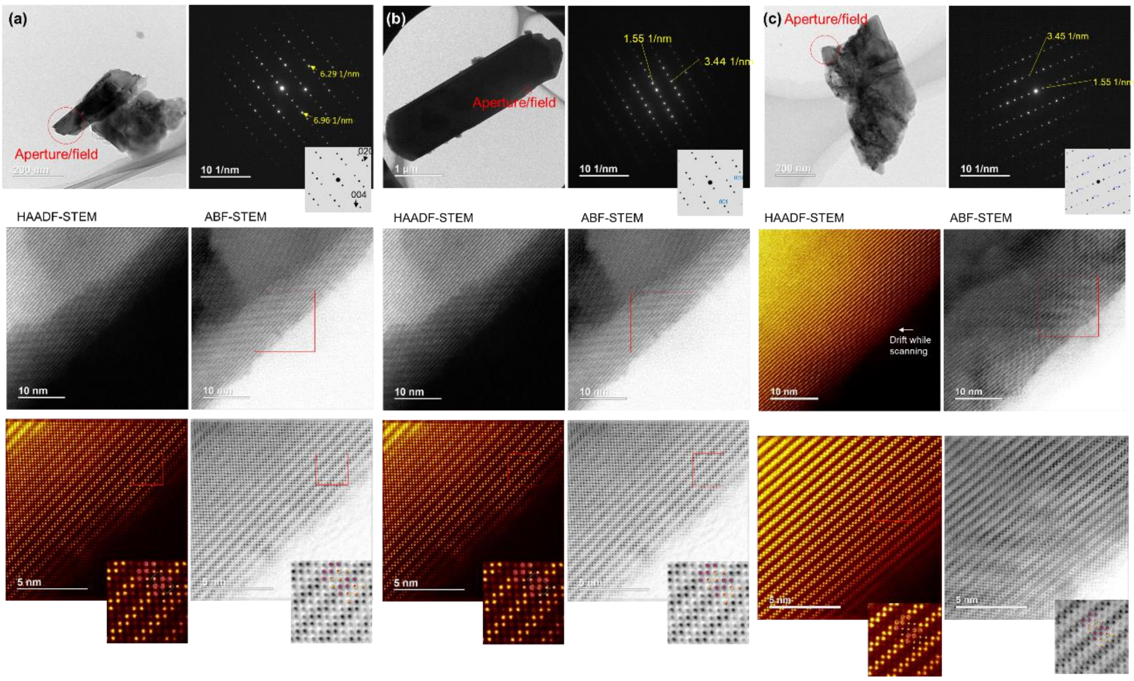

Figure S8. STEM image along the [100] zone axis for (a) NMO, (b) NMCO-2, and (c) NMZO

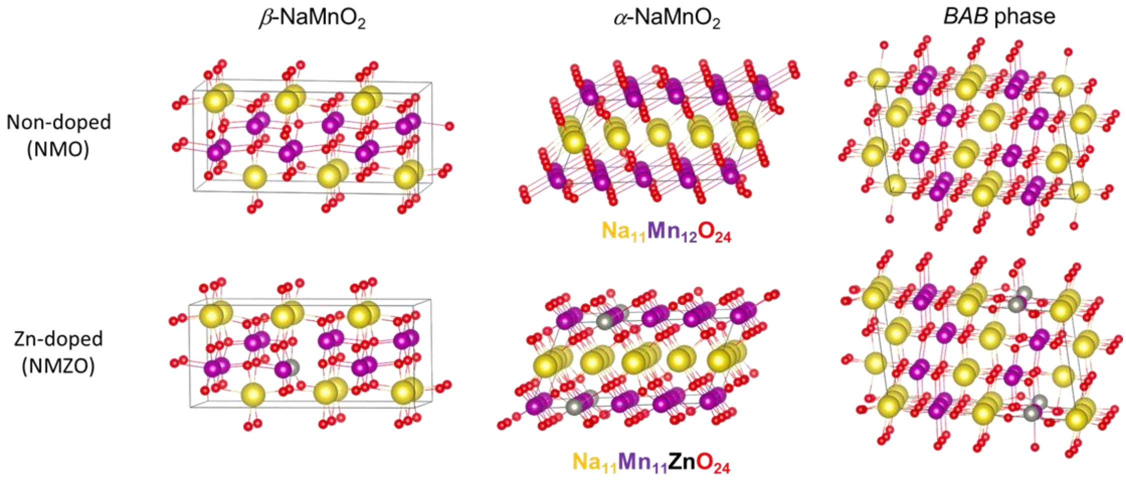

	α-phase	β-phase	***BAB***-phase
ΔE(NMO) (meV/f.u)	0	31	29
ΔE(NMZO) (meV/f.u)	0	53	36

Figure S9. Relative energy of α, β and BAB phases for NMO and NMZO obtained by GGA+U calculations.

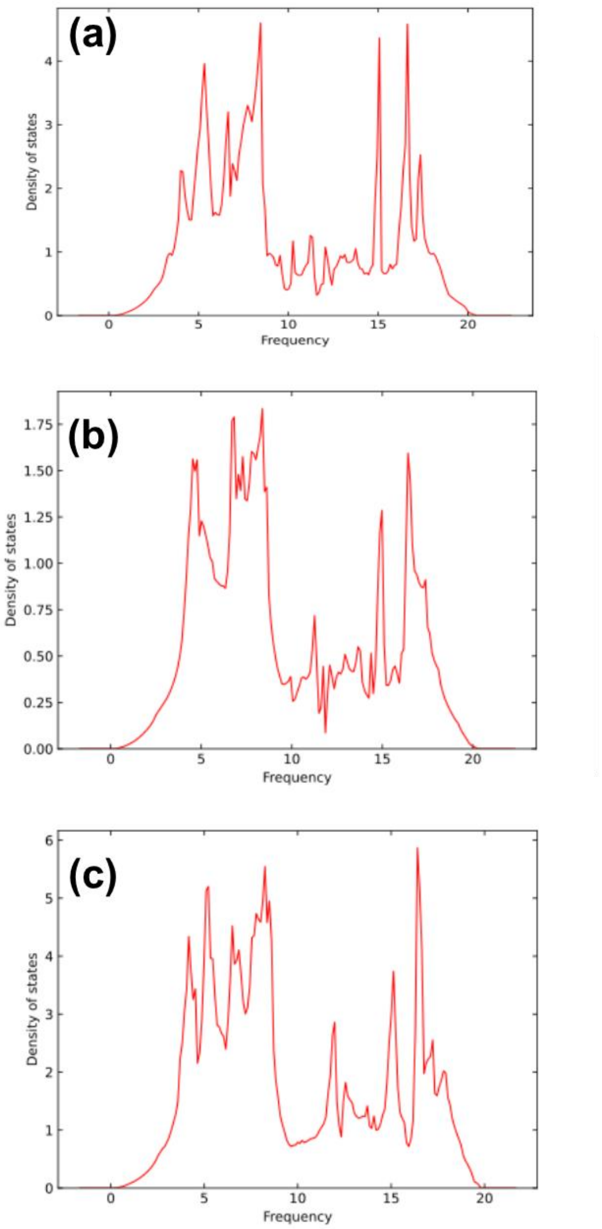

Figure S10. Phonon density of states of (a) α-, (b) β- and (c) *BAB*-phases of $NaMnO_2$

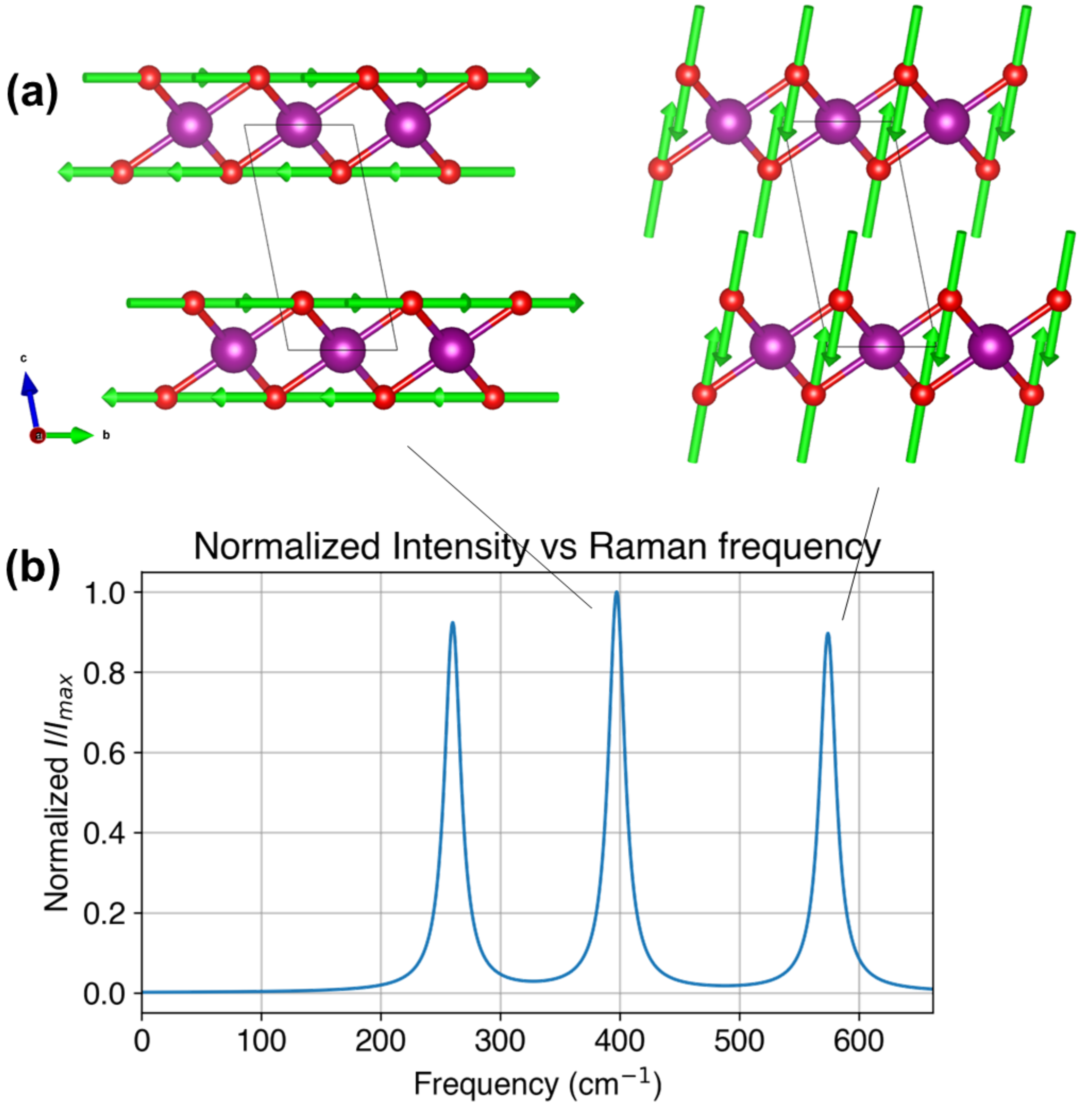

Figure S11. (a) Main Raman modes for α–phase, (b) Raman spectrum for the ideal α–$NaMnO_2$.

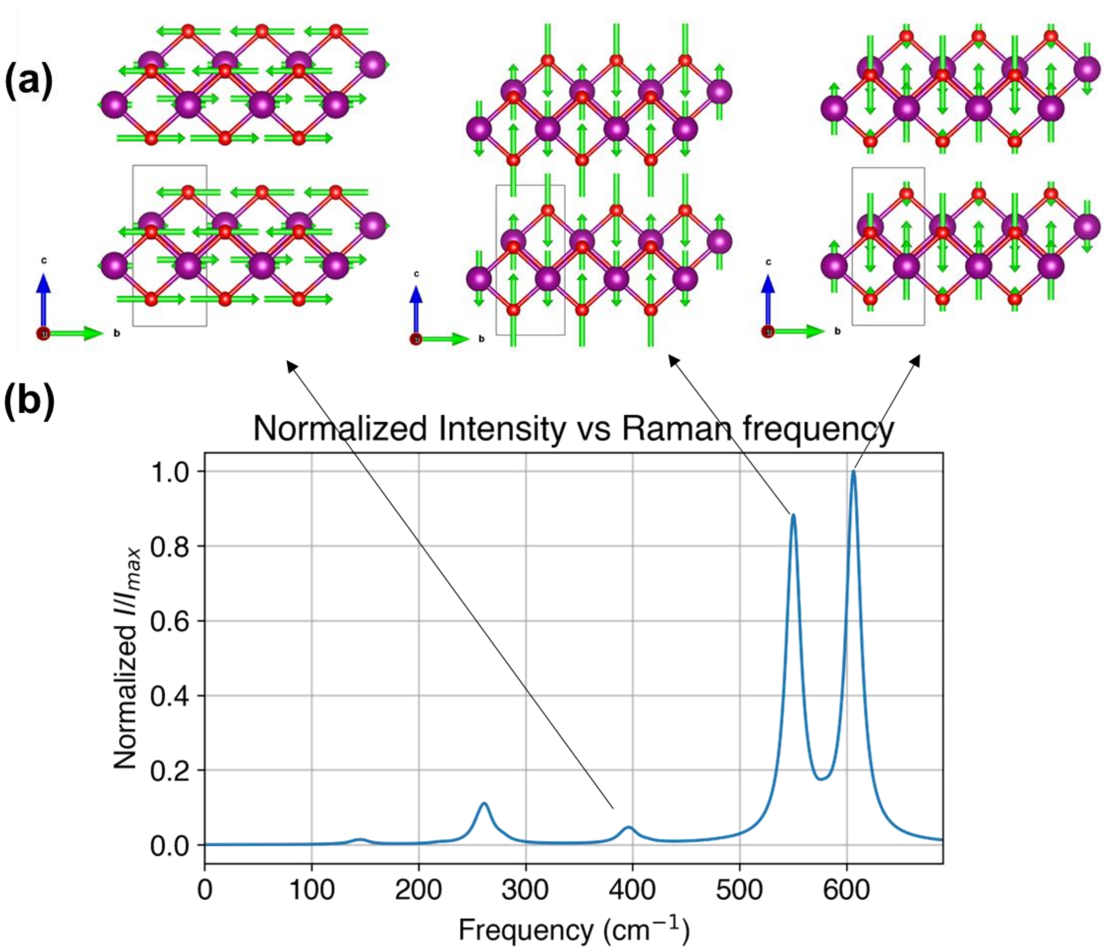

Figure S12. (a) Main Raman modes for *BAB*–phase, (b) Raman spectrum for the ideal *BAB*–$NaMnO_2$.

Table S3. Simulated Raman active modes with normalized intensity of β-$NaMnO_2$ phase

ν(cm^{-1})	Ir. Rep.	I/I_{max}
138.88	B2g	0.00
149.19	B3g	0.02
163.55	Ag	0.01
181.15	B2g	0.00
237.39	B3g	0.02
260.29	B3g	0.05
273.83	Ag	0.01
**399.36**	**B2g**	**0.02**
403.07	B3g	0.01
487.78	B2g	0.00
**545.05**	**Ag**	**0.75**
**614.07**	**Ag**	**1.00**

Table S4. Simulated Raman active modes with normalized intensity of α-$NaMnO_2$ phase

ν(cm^{-1})	Ir. Rep.	I/I_{max}
163.32	Bu	0.000
174.82	Au	0.000
226.89	Bu	0.000
**260.18**	**Ag**	**0.924**
290.85	Bu	0.000
**397.23**	**Bg**	**1.000**
464.59	Au	0.000
**573.89**	**Ag**	**0.899**
578.19	Bu	0.000

Table S5. Simulated Raman active modes with normalized intensity of *BAB*-$NaMnO_2$ phase

ν(cm^{-1})	Ir. Rep.	I/I_{max}
122.78	None	0.00
126.85	None	0.00
129.10	None	0.00
140.79	None	0.01
146.07	None	0.00
147.63	Ag	0.01
163.13	Bu	0.00
173.92	None	0.00
179.31	Bg	0.00
180.56	None	0.00
180.95	None	0.00
217.86	Bu	0.00
219.50	Ag	0.00
256.16	Ag	0.03
262.09	Ag	0.09
275.34	Bu	0.00
278.01	Ag	0.01
289.23	Bu	0.00
290.46	Bu	0.00
392.44	None	0.02
393.30	None	0.00
**398.19**	**None**	**0.03**
410.61	None	0.00
413.19	None	0.00
465.44	Au	0.00
481.06	Bg	0.00
482.73	Au	0.00
**550.16**	**Ag**	**0.88**
559.30	Bu	0.00
578.65	Ag	0.03
582.48	Bu	0.00
598.80	Bu	0.00
606.35	Ag	1.00

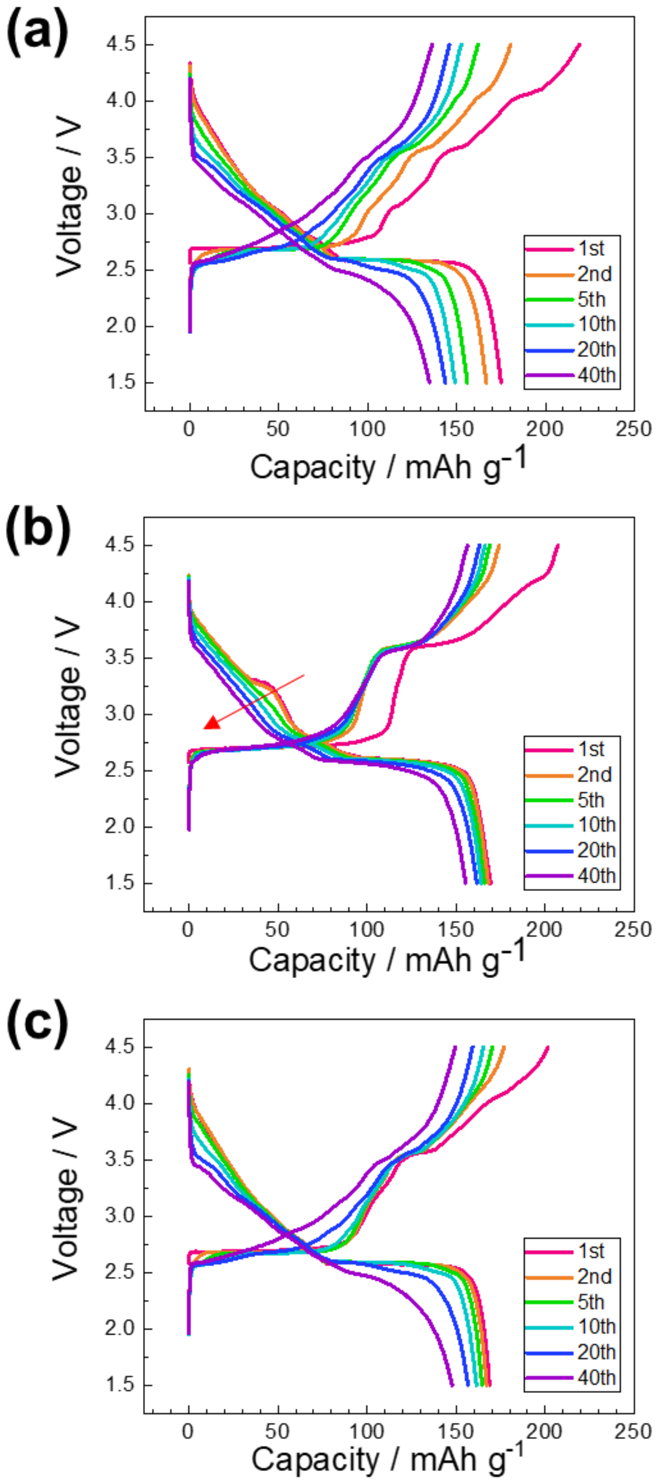

Figure S13. Galvanostatic charge/discharge curves of (a) NMO, (b) NMCO, and (c) NMZO at a rate of C/20 (≈ 12 mA g^{-1}).

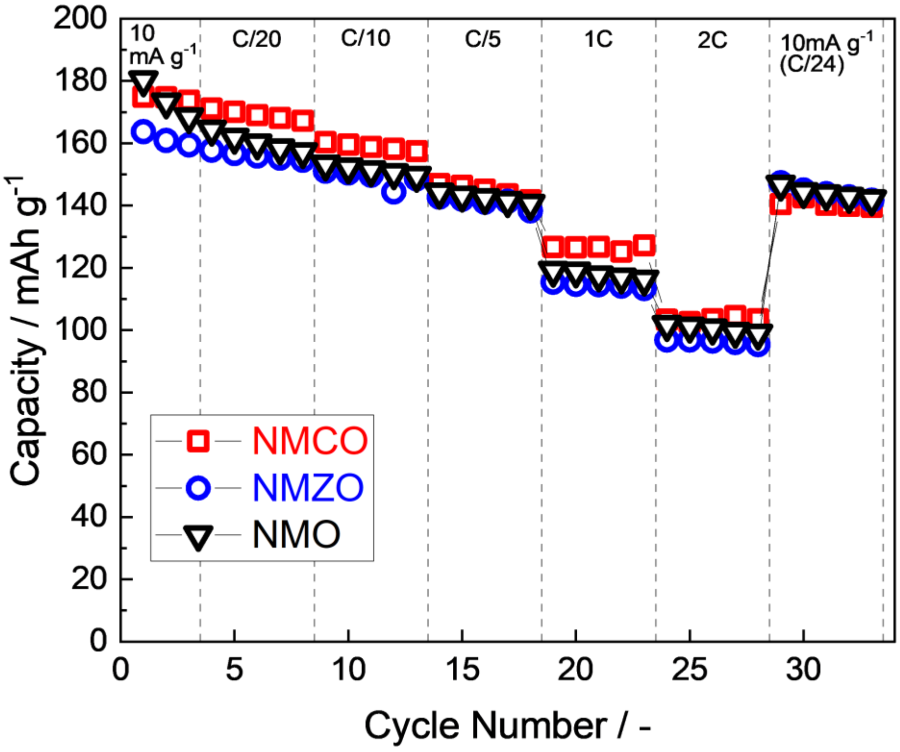

Figure S14. Discharge rate capacity with charge rate at C/20 (≈ 12 mA g^{-1}).

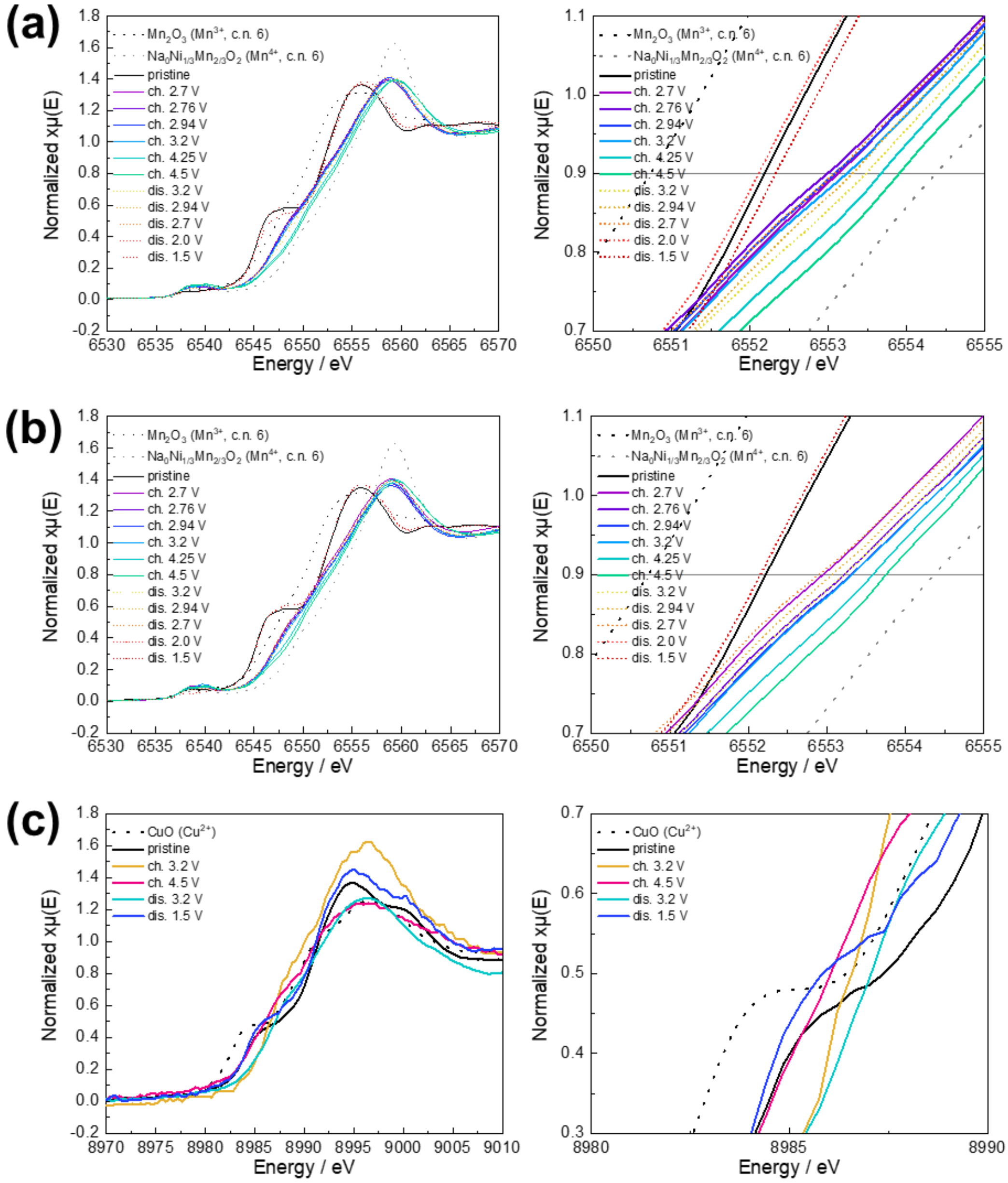

Figure S15. (a) Mn *K*-edge spectra for NMO, (b) Mn *K*-edge spectra for NMCO, and (c) Cu *K*-edge spectra for NMCO.

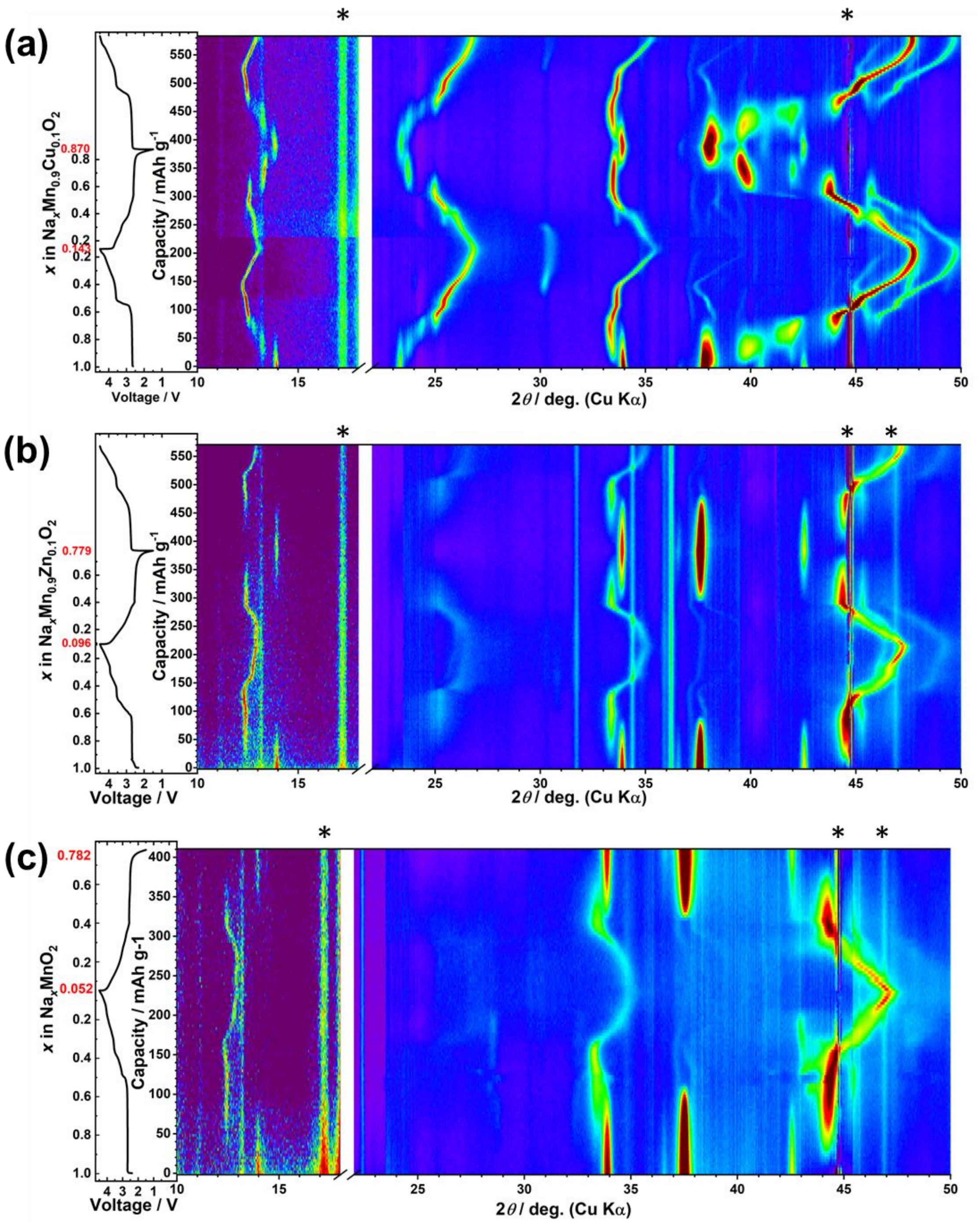

Figure S16. Operando XRD heat map upon the 1st charge, 1st discharge and 2nd charge for (a) NMCO and (b) NMZO, (c) upon 1st charge and discharge for NMO. Asterisks indicate the residual background after subtraction.

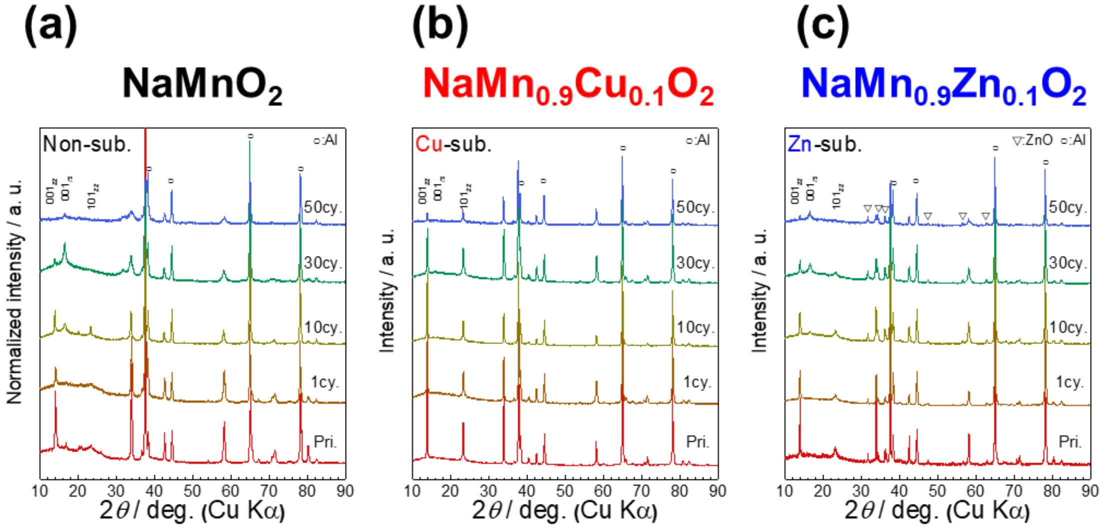

Figure S17. Ex-situ XRD patterns of 1-, 10-, 30-, and 50-cycled (a) $NaMnO_2$, (b) $NaMn_{0.9}Cu_{0.1}O_2$, and (c) $NaMn_{0.9}Zn_{0.1}O_2$. Each electrode was kept at 2.0 V.

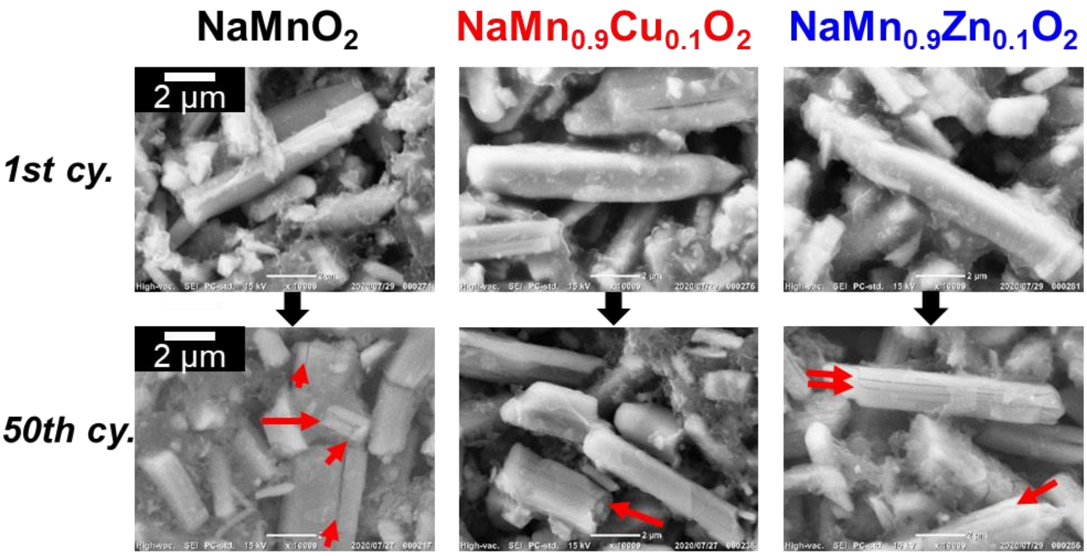

Figure S18. Ex-situ SEM images of the electrodes of $NaMn_{0.9}Me_{0.1}O_2$ (Me = Mn, Cu, Zn). Each electrode was kept at 2.0 V.

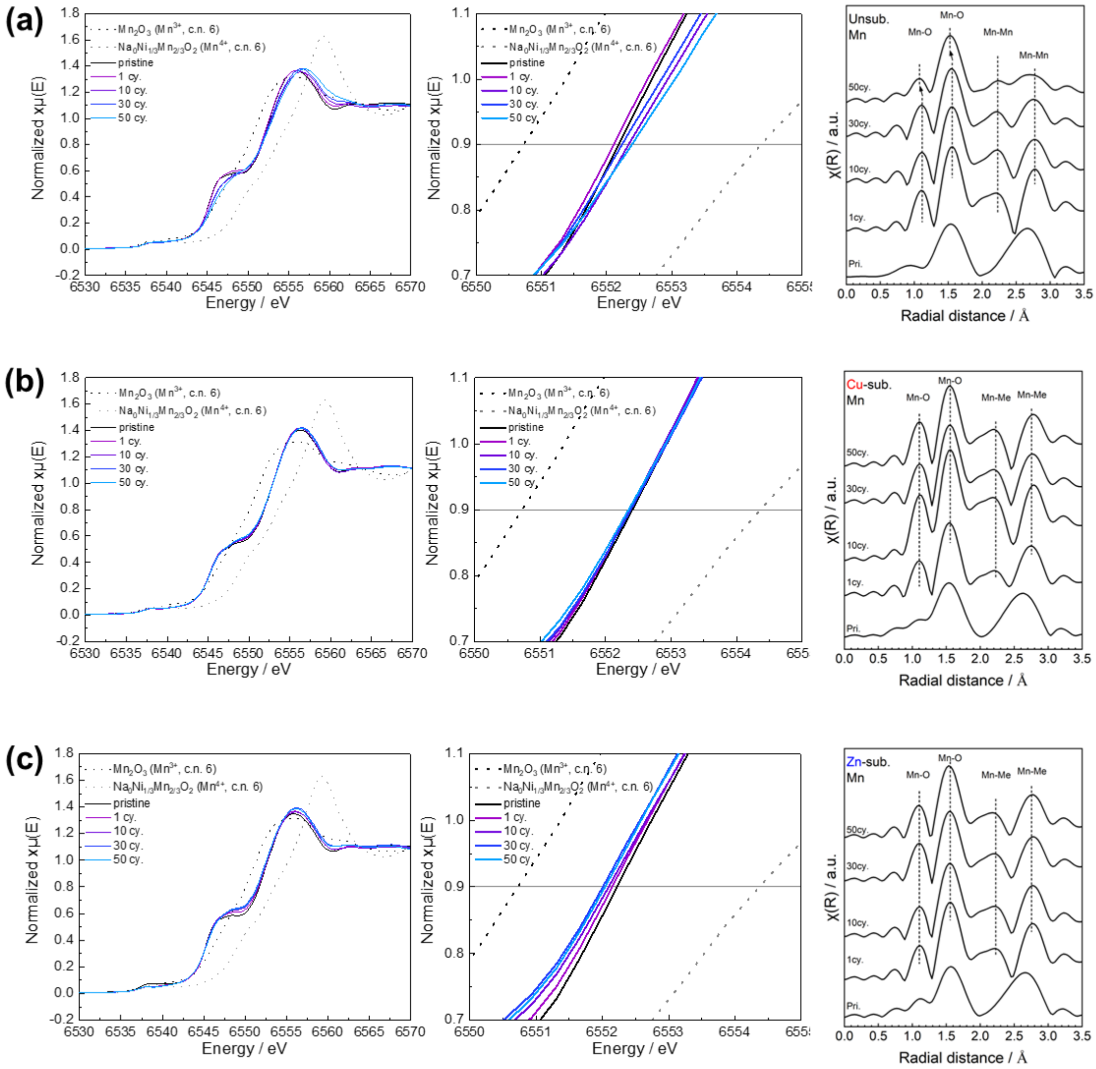

Figure S19. Ex-situ Mn K-edge spectra during cycling for (a) NMO, (b) NMCO and (c) NMZO

In all samples, the shoulder feature around 6547 eV progressively diminished with cycling. This tendency was most pronounced in NMO, followed by NMZO and then NMCO. Since this shoulder is associated with the Jahn–Teller distortion of the MnO_6 octahedra, the observed suppression in NMO implies an increase in Mn^{4+} content and a corresponding reduction in Jahn–Teller distortion due to progressive structural disorder upon cycling. According to previous reports, the MnO_6 octahedra in the zigzag-layered β-phase exhibit greater distortion than those in the α-phase. Considering the concurrent increase in α-phase content observed in the ex situ XRD results, the reduction of the XANES shoulder feature can be attributed to the gradual structural transition toward the α-type configuration with less Jahn–Teller distortion.

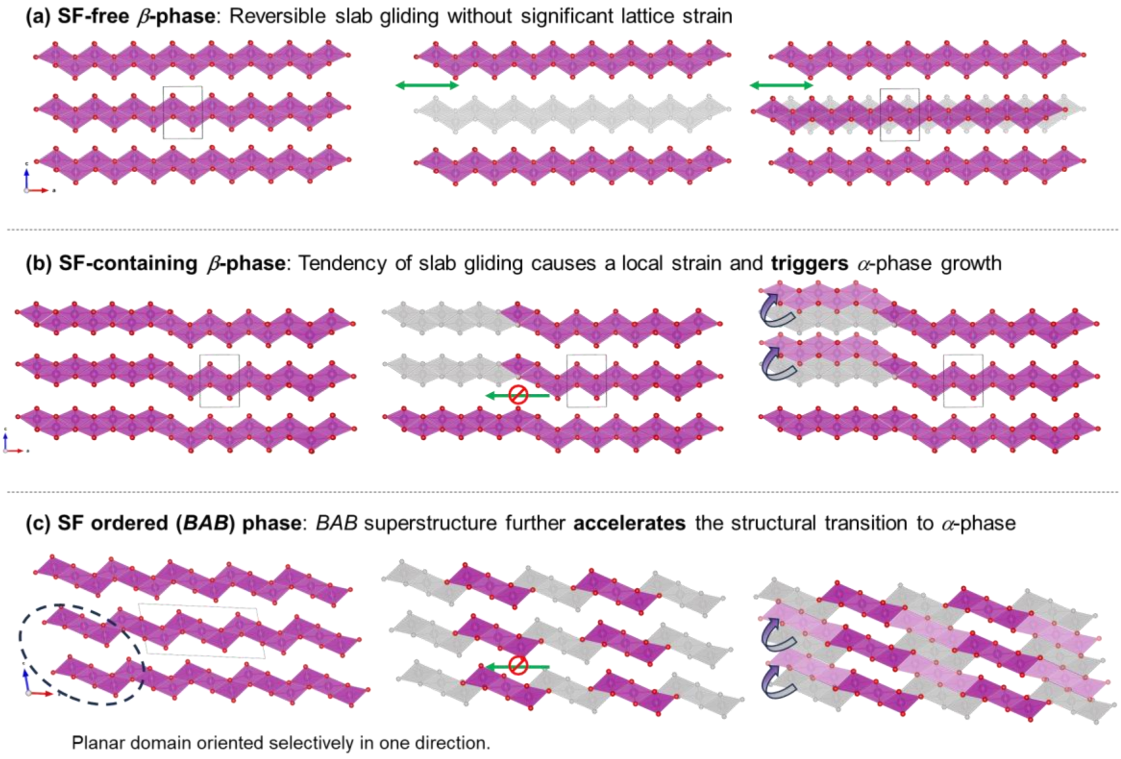

Figure S20. Schematic illustration of the structural changes to α-phase from (a) SF-free β-phase, (b) SF-containing β-phase, and (c) SF-ordered β-phase.

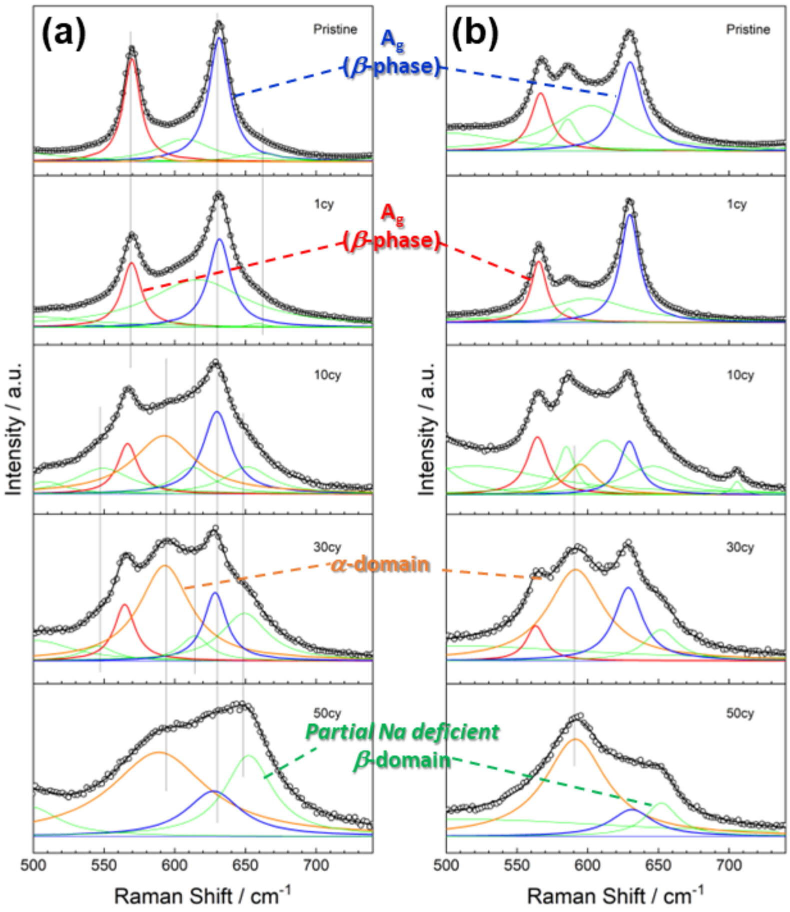

Figure S21. Fitting results of ex-situ Raman spectra during cycling for (a) NMO and (b) NMZO.

Reference

(1) Kubota, K.; Miyazaki, M.; Kim, E. J.; Yoshida, H.; Barpanda, P.; Komaba, S. Structural Change Induced by Electrochemical Sodium Extraction from Layered O′3-$NaMnO_2$. *J. Mater. Chem. A* **2021**, *9* (47), 26810–26819. https://doi.org/10.1039/D1TA05390F.

(2) Croguennec, L.; Deniard, P.; Brec, R.; Lecerf, A. Nature of the Stacking Faults in Orthorhombic LiMnO2. *J. Mater. Chem.* **1997**, *7* (3), 511–516. https://doi.org/10.1039/a604947h.

(3) Kawaguchi, S.; Takemoto, M.; Osaka, K.; Nishibori, E.; Moriyoshi, C.; Kubota, Y.; Kuroiwa, Y.; Sugimoto, K. High-Throughput Powder Diffraction Measurement System Consisting of Multiple MYTHEN Detectors at Beamline BL02B2 of SPring-8. *Review of Scientific Instruments* **2017**, *88* (8), 085111. https://doi.org/10.1063/1.4999454.

(4) Izumi, F.; Momma, K. Three-Dimensional Visualization in Powder Diffraction. *Solid State Phenomena* **2007**, *130*, 15–20. https://doi.org/10.4028/www.scientific.net/SSP.130.15.

(5) Momma, K.; Izumi, F. VESTA 3 for Three-Dimensional Visualization of Crystal, Volumetric and Morphology Data. *J Appl Cryst* **2011**, *44* (6), 1272–1276. https://doi.org/10.1107/S0021889811038970.

(6) Masese, T.; Miyazaki, Y.; Rizell, J.; Kanyolo, G. M.; Chen, C.-Y.; Ubukata, H.; Kubota, K.; Sau, K.; Ikeshoji, T.; Huang, Z.-D.; Yoshii, K.; Takahashi, T.; Ito, M.; Senoh, H.; Hwang, J.; Alshehabi, A.; Matsumoto, K.; Matsunaga, T.; Fujii, K.; Yashima, M.; Shikano, M.; Tassel, C.; Kageyama, H.; Uchimoto, Y.; Hagiwara, R.; Saito, T. Mixed Alkali-Ion Transport and Storage in Atomic-Disordered Honeycomb Layered NaKNi2TeO6. *Nat Commun* **2021**, *12* (1), 4660. https://doi.org/10.1038/s41467-021-24694-5.

(7) Saito, M.; Kimoto, K.; Nagai, T.; Fukushima, S.; Akahoshi, D.; Kuwahara, H.; Matsui, Y.; Ishizuka, K. Local Crystal Structure Analysis with 10-Pm Accuracy Using Scanning Transmission Electron Microscopy. *Journal of Electron Microscopy* **2009**, *58* (3), 131–136. https://doi.org/10.1093/jmicro/dfn023.

(8) Kresse, G. *Ab Initio* Molecular Dynamics for Liquid Metals. *Phys. Rev. B* **1993**, *47* (1), 558–561. https://doi.org/10.1103/PhysRevB.47.558.

(9) Kresse, G. From Ultrasoft Pseudopotentials to the Projector Augmented-Wave Method. *Phys. Rev. B* **1999**, *59* (3), 1758–1775. https://doi.org/10.1103/PhysRevB.59.1758.

(10) Perdew, J. P. Generalized Gradient Approximation Made Simple. *Phys. Rev. Lett.* **1996**, *77* (18), 3865–3868. https://doi.org/10.1103/PhysRevLett.77.3865.

(11) Jain, A. Formation Enthalpies by Mixing GGA and GGA + U Calculations. *Phys. Rev. B* **2011**, *84* (4). https://doi.org/10.1103/PhysRevB.84.045115.

(12) Luong, H. D.; Dinh, V. A.; Momida, H.; Oguchi, T. Insight into the Diffusion Mechanism of Sodium Ion–Polaron Complexes in Orthorhombic P2 Layered Cathode Oxide NaxMnO2. *Phys. Chem. Chem. Phys.* **2020**, *22* (32), 18219–18228. https://doi.org/10.1039/D0CP03208E.

(13) Dudarev, S. L. Electron-Energy-Loss Spectra and the Structural Stability of Nickel Oxide: An LSDA U Study. *Phys. Rev. B* **1998**, *57* (3), 1505–1509. https://doi.org/10.1103/PhysRevB.57.1505.

(14) Grimme, S.; Antony, J.; Ehrlich, S.; Krieg, H. A Consistent and Accurate *Ab Initio* Parametrization of Density

Functional Dispersion Correction (DFT-D) for the 94 Elements H-Pu. *The Journal of Chemical Physics* **2010**, *132* (15), 154104. https://doi.org/10.1063/1.3382344.

(15) Luong, H. D.; Pham, T. D.; Morikawa, Y.; Shibutani, Y.; Dinh, V. A. Diffusion Mechanism of Na Ion–Polaron Complex in Potential Cathode Materials NaVOPO4 and VOPO4 for Rechargeable Sodium-Ion Batteries. *Phys. Chem. Chem. Phys.* **2018**, *20* (36), 23625–23634. https://doi.org/10.1039/C8CP03391A.

(16) Luong, H. D.; Xu, C.; Jalem, R.; Tateyama, Y. Evaluation of Battery Positive-Electrode Performance with Simultaneous Ab-Initio Calculations of Both Electronic and Ionic Conductivities. *Journal of Power Sources* **2023**, *569*, 232969. https://doi.org/10.1016/j.jpowsour.2023.232969.

(17) Togo, A.; Chaput, L.; Tadano, T.; Tanaka, I. Implementation Strategies in Phonopy and Phono3py. *J. Phys.: Condens. Matter* **2023**, *35* (35), 353001. https://doi.org/10.1088/1361-648X/acd831.

(18) Skelton, J. M.; Burton, L. A.; Jackson, A. J.; Oba, F.; Parker, S. C.; Walsh, A. Lattice Dynamics of the Tin Sulphides SnS2, SnS and Sn2S3: Vibrational Spectra and Thermal Transport. *Phys. Chem. Chem. Phys.* **2017**, *19* (19), 12452–12465. https://doi.org/10.1039/C7CP01680H.

(19) Kumakura, S.; Tahara, Y.; Kubota, K.; Chihara, K.; Komaba, S. Sodium and Manganese Stoichiometry of P2-Type $Na_{2/3}$ MnO_2. *Angew Chem Int Ed* **2016**, *55* (41), 12760–12763. https://doi.org/10.1002/anie.201606415.

(20) Kumakura, S.; Tahara, Y.; Sato, S.; Kubota, K.; Komaba, S. P′2-$Na_{2/3}$ $Mn_{0.9}$ $Me_{0.1}$ O_2 (Me = Mg, Ti, Co, Ni, Cu, and Zn): Correlation between Orthorhombic Distortion and Electrochemical Property. *Chem. Mater.* **2017**, *29* (21), 8958–8962. https://doi.org/10.1021/acs.chemmater.7b02772.
